# TIM3^+^ breast cancer cells license immune evasion during micrometastasis outbreak

**DOI:** 10.1016/j.ccell.2025.06.015

**Published:** 2025-08-11

**Authors:** Catalina Rozalén, Irene Sangrador, Silvia Avalle, Sandra Blasco-Benito, Panagiota Tzortzi, María Sanz-Flores, José Ángel Palomeque, Pau Torren-Duran, Mariona Dalmau, Helena Brunel, Albert Coll-Manzano, Iván Pérez-Núñez, Tamara Martos, Sonia Servitja, Sandra Pérez-Buira, José Ignacio Chacón, Ángel Guerrero-Zotano, Eduardo Martínez de Dueñas, Yolanda Guillén, Laura Comerma, Begoña Bermejo, Anna Bigas, María Casanova-Acebes, Anna Alemany, Federico Rojo, Joan Albanell, Toni Celià-Terrassa

**Affiliations:** 1 Cancer Research Program, Hospital del Mar Research Institute, Barcelona, Spain; 2 Pathology Department, Hospital del Mar, Barcelona, Spain; 3 Universitat Pompeu Fabra, Barcelona, Spain; 4 Centro de Investigación Biomédica en Red de Oncología (CIBERONC-ISCIII), Madrid, Spain; 5 Medical Oncology Department, Hospital del Mar, Barcelona, Spain; 6 GEICAM Spanish Breast Cancer Group, Madrid, Spain; 7 Pathology Department, IIS, Fundación Jimenez Diaz, UAM, Madrid, Spain; 8 Hospital Universitario de Toledo, Toledo, Spain; 9 Instituto Valenciano Oncología (IVO), Valencia, Spain; 10 Consorcio Hospitalario Provincial de Castellón, Castellón, Spain; 11 Medical Oncology Department, Hospital Clínico Universitario, Medicine Department, Universidad de Valencia, INCLIVA, Valencia, Spain; 12 Josep Carreras Leukemia Research Institute, Badalona, Spain; 13 Spanish National Cancer Research Center (CNIO), Madrid, Spain; 14 Department of Anatomy & Embryology, Leiden University Medical Center, Leiden, the Netherlands; 15 The Novo Nordisk Foundation Center for Stem Cell Medicine (reNEW), Leiden, the Netherlands

**Keywords:** micrometastasis, cancer immunoediting, metastasis, breast cancer, TIM3, immune-evasion, EMT, stemness, γδ T cells, TIM3 blockade

## Abstract

In metastasis, the dynamics of tumor-immune interactions during micrometastasis remain unclear. Identifying the vulnerabilities of micrometastases before outbreaking into macrometastases can reveal therapeutic opportunities for metastasis. Here, we report a function of T cell immunoglobulin and mucin domain 3 (TIM3) in tumor cells during micrometastasis using breast cancer (BC) metastasis mouse models. TIM3 is highly upregulated in micrometastases, promoting survival, stemness, and immune escape. TIM3^+^ tumor cells are specifically selected during early seeding of micrometastasis. Mechanistically, TIM3 increases β-catenin/interleukin-1β (IL-1β) signaling, leading to stemness and immune-evasion by inducing immunosuppressive γδ T cells and reducing CD8 T cells during micrometastasis. Clinical data confirm increased TIM3^+^ tumor cells in BC metastasis and TIM3^+^ tumor cells as a biomarker of poor outcome in BC patients. (Neo)adjuvant TIM3 blockade reduces the metastatic seeding and incidence in preclinical models. These findings unveil a specific mechanism of micrometastasis immune-evasion and the potential use of TIM3 blockade for subclinical metastasis.

## Highlights

• Immune pressure at distant organs selects TIM3^+^ tumor cells during micrometastasis
• TIM3^+^ tumor cells exhibit stemness, EMT, and immune-evasive features
• TIM3 in tumor cells is a biomarker of relapse and poor prognosis in BC patients
• (Neo)adjuvant TIM3 blockade prevents metastatic seeding and metastasis initiation

## Graphical abstract

**Figure:**
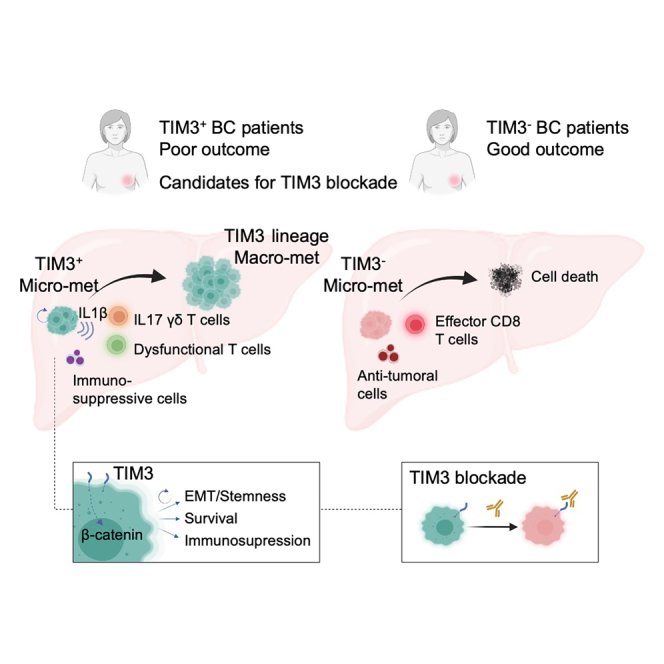

## Teaser

Rozalén et al. report that TIM3 in breast cancer drives a specific mechanism of immune escape during micrometastasis. TIM3^+^ tumor cells exhibit stemness/EMT features and promote immune-evasion by inducing immunosuppressive γδ T cells. TIM3 is a biomarker of poor outcome, and its blockade targets metastasis initiating cells during micrometastasis.

## Introduction

Metastatic breast cancer is not curable with current therapies and accounts for nearly 700,000 deaths every year.^1^ Adjuvant and neoadjuvant therapeutic strategies aim to prevent and/or eradicate micrometastatic disease and hence progression to overt metastatic disease. However, micrometastasis immunity is poorly understood due to preclinical and clinical challenges. In order to improve these strategies, we need a comprehensive understanding of the biology of small early micrometastasis and their tumor microenvironment.

During metastasis, most of the disseminated tumor cells (DTCs) fail to adapt to distant tissue conditions upon arrival and die, resulting in a selection of the fittest cells. Among these hurdles, the immune system’s anti-tumor activity is one of the main barriers for metastatic colonization.^2^^,^^3^ Then, metastasis-initiating cells (MICs) are a selection of few tumor cells with distinctive properties enabling the seeding and growth in distant sites.^4^^,^^5^ Tumor phenotypes considered highly metastatic or MICs, such as cancer stem cell (CSC) and epithelial-to-mesenchymal transition (EMT)-like phenotypes,^4^^,^^5^ have also been described as immune-evasive in other studies.^6^^,^^7^^,^^8^^,^^9^ The immune landscape of distant organs impose immune pressure on tumor cells engaging a process of tumor-immune coevolution and selection of immune-evasive tumor cells, in a process called cancer immunoediting.^10^ Functional and genomic studies have reported the existence of immunoediting during metastasis in humans and experimental mouse models.^11^^,^^12^^,^^13^ Moreover, different organs display different immune requisites and pressures. For instance the liver metastasis immunity is of major interest due to its tolerogenic immune cell populations, which make these metastases more resistant to immunotherapy than other organs.^14^ Yet, the tumor phenotype dynamics overcoming the immune pressure require further investigation during micrometastasis.

TIM3 (T cell immunoglobulin and mucin domain 3), also known as HAVCR2, is a cellular receptor typically expressed in immune cells where it functions as an immune checkpoint receptor. In particular, it is expressed in interferon gamma (IFN-γ) activated T cells and is important in T cell dysfunction in cancer^15^. Therefore, TIM3 blockade has been proposed as an immune-checkpoint inhibitor (ICI), and there are ongoing clinical trials in advanced metastatic disease for acute myeloid leukemia (AML),^16^ lung cancer,^17^ and melanoma.^18^ However, TIM3 expression is not only restricted to immune cells, and recent studies have reported TIM3 expression in normal epithelial cells and tumor cells,^19^ including leukemia stem cells^20^ and diffuse intrinsic pontine glioma (DIPG)^21^ triggering AKT and β-catenin signaling. Importantly, the biology of TIM3 and its potential therapeutic effects on metastatic tumor cells have not been explored, nor has its use in time-tailored therapies to block metastasis initiation. Here, we show how TIM3 expression in breast cancer cells is a biomarker of poor outcome and high-risk of relapse in breast cancer (BC) patients and plays a unique role in MICs specific of micrometastasis, including survival, stemness, and immune-evasion.

## Results

### Modeling experimental metastasis immunoediting

**Figure 1:**
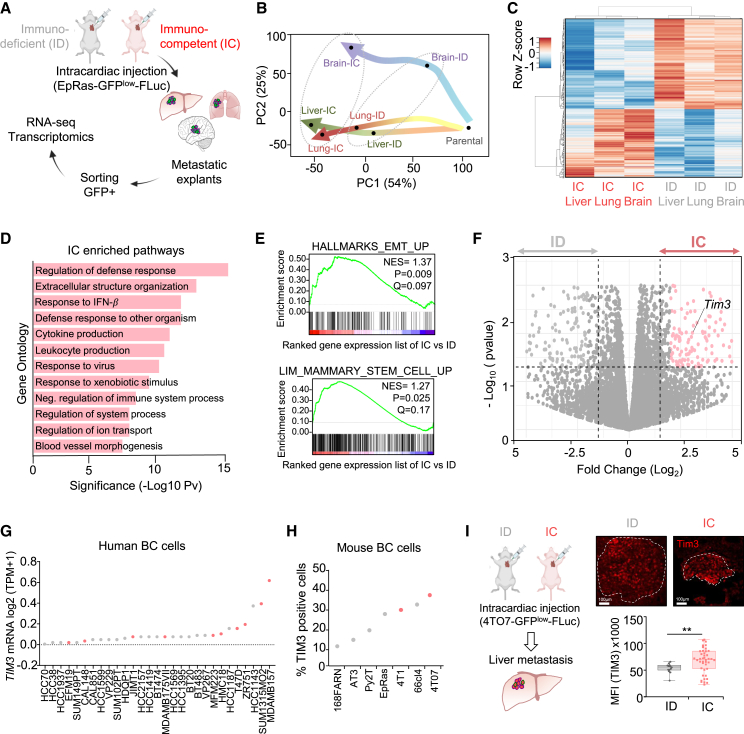
Metastasis immune pressure positively selects TIM3^+^ metastatic cells (A) Experimental design using immunocompetent (IC) Balb/c mice and immunodeficient (ID) NOD scid gamma (NSG) mice for the assessment of metastatic immune pressure. (B) Principal-component analysis (PCA) of the RNA-seq from EpRas cells isolated from three organ (lung, liver, and brain) metastatic samples in IC and ID hosts. (C) Unsupervised hierarchical clustering from lung, liver and brain metastasis (*Z* score) in ID and IC hosts (*n* = 3 IC and *n* = 3 ID independent biological replicates). (D) Gene ontology enrichment analysis of top 50 upregulated genes in all organs from IC mice. (E) GSEA of indicated gene lists with the ranked gene expression list of IC vs. ID in all organ samples. (F) Volcano plot of gene expression in all organs comparing IC and ID mice samples (*n* = 3 independent biological replicates). (G) Dot plot representing *TIM3* expression of human breast cancer cell lines from the CCLE. Primary tumor-derived cells (gray) and metastasis-derived cells (pink). (H) Dot plot representing TIM3 protein levels measured by flow cytometry of mouse breast cancer cell lines with metastatic potential in experimental models. Color code as (G). (I) TIM3 immunofluorescence of liver tissue metastasis derived from 4T07 intracardiac injection in ID and IC mice. Representative image of TIM3 immunofluorescence. Scale bars, 100 μm. Dashed line delineates metastasis tissue. Boxplot quantification of TIM3 staining mean fluorescent intensity in from 5 independent mice (*n* = 5 independent biological replicates). Data represent mean ± SEM. Statistical significance; ^∗^*p* < 0.05, ^∗∗^*p* < 0.01, and ^∗∗∗^*p* < 0.001, by unpaired Student’s t test. Also see Figure S1 and Table S1.

To measure the immune pressure executed on tumor cells during metastatic colonization at distant organs, we conducted comparative experimental metastasis in (NOD.Cg-*Prkdc*^*scid*^ *Il2rg*^*tm1Wjl*^/SzJ) NOD scid gamma (NSG) immunodeficient (ID) and Balb/c immunocompetent (IC) mice. We used EpRas breast tumor-transformed mouse cells, which have never been previously immunoedited as they are not derived from tumor tissues *in vivo*,^22^ and are an established model of breast cancer metastasis.^23^^,^^24^^,^^25^ First, EpRas-FLuc-GFP cells were selected with low-GFP expression (Figure S1A) to avoid immunogenicity as previously suggested,^26^ and transplanted with low cell number via intracardiac injection (i.c.) for systemic delivery in ID and IC mice, monitored by bioluminescence imaging (BLI) (Figures 1A and S1B). As expected, the metastatic growth was reduced in IC mice compared to ID mice indicating the selective effect of the host immune system (Figure S1B). Twenty days after injection, lung, liver, and brain metastasis were dissected, detected by BLI *ex vivo* (Figure S1C) and tumor cells were isolated by flow cytometry based on GFP. Next, RNA sequencing (RNA-seq) analysis of ID and IC-derived metastatic tumor cells revealed differences in the transcriptomic profiles based on the immunoediting selection suffered in IC hosts. RNA-seq data validated the specificity of the tumor cell isolation methodology since no immune-exclusive genes were detected, such as *Cd45* (Figure S1D). Principal-component analysis (PCA) and unsupervised hierarchical clustering showed how the transcriptomic profiles of metastases from IC hosts were differentially clustered from ID hosts (Figures 1B and 1C). The IC and ID-derived metastasis samples clustered independently of the metastatic site, indicating that the immune pressure is a dominant selective factor of tumor cell traits during metastasis (Figures 1B and 1C).

Looking for commonalities in the 3 metastatic organs (brain, lung, and liver) IC vs. ID, gene ontology (GO) analysis showed that IC-derived metastatic cells were enriched in pathways related to negative immune system regulation (GO:002683) (Figures 1D, S1E, and S1F), validating our experimental design to measure metastatic immune pressure. IC-derived metastasis showed enrichment in stem cell-like pathways including LIM_mammary stem cells^27^ and the hallmarks of EMT (MSigDB-M5930) datasets by gene set enrichment analysis (GSEA)^28^ (Figure 1E). These results suggested that immunoedited metastatic cells are enriched in immune-evasive and EMT stem cell-like properties, which are aligned with computational studies associating stemness and immune-evasion in clinical metastasis.^29^^,^^30^ Among the upregulated genes in immunoedited metastases, we focused on potential druggable targets. Strikingly, we found high levels of *Tim3* expression in tumor cells in all organs (Figure 1F and Table S1), particularly in liver metastasis (Figure S1G). This was unexpected since TIM3 is a T cell immunoglobulin mucin family member typically expressed in immune cells^31^ and functioning as an immune-checkpoint receptor in lymphocytes.^32^ Its role in epithelial cells has been underestimated and only recently few reports have started to explore it.^19^^,^^21^

Using the cancer cell line encyclopedia (CCLE), we checked that human breast cancer cells indeed express *TIM3* with high variability across different cell lines (Figure 1G). To choose the best syngeneic BC metastasis models to interrogate TIM3 functions *in vivo*, we analyzed TIM3 protein levels by flow cytometry in mouse BC cells (Figure 1H). Similarly, mouse BC cells express TIM3, and detected the highest levels of TIM3 in 66cl4, 4T07, and 4T1 cells (Figure 1H), which are triple-negative breast cancer (TNBC) metastasis models. We selected 4T07 with high TIM3 levels to perturb TIM3 in functional experimental metastasis *in vivo*. 4T07 cells cause multi-organ metastasis when injected i.c.^33^ Similar to EpRas, the i.c. injection of 4T07-FLuc-GFP cells in IC and ID hosts showed less metastases in IC conditions as expected due to immune pressure (Figure S1H), and thus increased levels of TIM3 in IC hosts (Figures 1I and S1I). Luciferase tissue staining validated the faithful monitoring of metastasis by BLI (Figure S1J).

### TIM3 promotes metastatic ability under immune pressure

**Figure 2:**
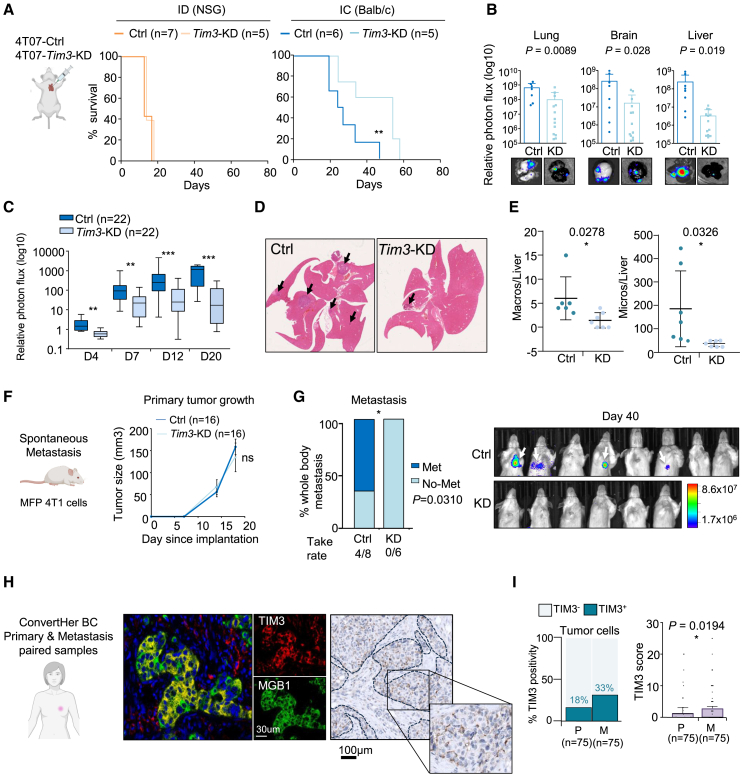
TIM3 drives breast cancer metastasis (A) Kaplan-Meier survival plot after intracardiac injection of 4T07 control cells versus *Tim3*-KD cells in ID (NSG) and IC (Balb/c) mice with the indicated conditions. Statistical analysis using Log rank (Mantel-Cox) test. (B) Relative photon flux BLI quantification of metastatic organs at day 16 after i.c. injection of 4T07 Ctrl and *Tim3*-KD cells. Data represents mean + SEM; dots represent independent biological replicates. (C) Relative photon flux BLI quantification of whole-body metastasis of 4T07 Ctrl and *Tim3*-KD in IC mice. Data represents mean + SEM. *n* = 22 independent biological replicates. (D) Hematoxylin-eosin staining of metastatic livers from unlabeled 4T07-Ctrl and -*Tim3*-KD cells. Arrows indicate metastatic lesions. (E) Quantification of micro- and macro-metastatic lesions from (D). (F) Mammary fat pad (MFP) injection of 4T1-Ctrl and -*Tim3*-KD cells in Balb/c mice. Data represents tumor growth by mean + SEM of *n* = 16 independent biological replicates. (G) Incidence of spontaneous metastasis at day 40 after primary tumor resection (day 20) of 4T1-Crtl and 4T1-*Tim3*-KD MFP injected mice. Individual BLI images from upper body. *n* = 8 Ctrl and *n* = 6 KD mice followed after resection. Statistical significance; ^∗^*p* < 0.05, ^∗∗^*p* < 0.01, and ^∗∗∗^*p* < 0.001, by Chi-square test. (H) Representative immunofluorescence of breast tumor cell marker Mamaglobulin-1 (MGB1) in red and TIM3 in green in human breast cancer tissue. Scale bars, 30 μm. Representative immunohistochemistry image of TIM3 showing tumor-epithelial cell staining. Dash line delineates the tumor areas. Scale bars, 100 μm. (I) Percentage of tumor TIM3-positive samples from primary (P) and metastatic (M) matched clinical samples (ConvertHER cohort). TIM3 score percentage in primary and paired-metastatic samples (right panel). *n* = 75 for each condition P and M. Data represented as mean ± SEM. Statistical significance; ^∗^*p* < 0.05, ^∗∗^*p* < 0.01, and ^∗∗∗^*p* < 0.001, by two-tailed Student’s t test in (B), (C), (E), and (I). Also see Figure S2.

In order to study the relevance of TIM3 during metastasis, we tested the metastatic ability of TIM3 gain- and loss-of-function in different mouse strains using 4T07, 4T1, and AT3 tumor cells, with Balb/c and C57BL/6 origin, respectively, with different immunity.^34^^,^^35^ The efficiency of KD and the overexpression (OE) of TIM3 in 4T07 was validated (Figure S2A). Systemic administration by intracardiac injection of 4T07-Fluc-GFP *Tim3*-KD (shRNA-86) cells significantly reduced their metastatic ability increasing the overall survival only in IC Balb/c hosts (Figures 2A and S2B). However, no significant KD effects were observed in ID hosts (Figures 2A and S2B), suggesting that TIM3 confers functional advantages in metastasis by overcoming immunosurveillance. The metastatic growth was reduced in all the organs, especially in the liver (Figure 2B). Additional experiments showed consistent reduced metastasis in *Tim3*-KD in IC hosts (Figure 2C) and also using multiple shRNAs (Figure S2C). In order to study the interference of the immunogenic GFP-Luc of our cell lines, we performed experiments with unlabeled 4T07-*Tim3*-KD cells which also reduced metastasis and increased mice survival compared to control cells (Figures 2D, 2E, and S2D). Consistently, AT3-*Tim3*-KD cells i.c. injected in IC C57BL/6 mice also showed extended survival than control cells, only in IC hosts (Figure S2E). Instead, 4T07-*Tim3*-OE showed increased metastasis and impaired survival compared to control mice (Figures S2F–S2H). Overall, these results demonstrate a selective immune-evasive pro-metastatic advantage of TIM3 in tumor cells.

Next, we used spontaneous experimental metastasis models by transplanting 4T1 metastatic cells into the mammary fat pad (MFP) followed by primary tumor resection upon 8 × 8 mm size and allow time to develop metastases. Interestingly, TIM3 did not affect the primary tumor growth; however, metastasis incidence in lung and liver organs was reduced by knocking-down *Tim3* in 4T1 cells (Figures 2F and 2G). Primary tumor proliferation was not affected by *Tim3*-KD in 4T07 cells either (Figures S2I and S2J). These data indicate that TIM3 leads a prominent functional role specific of metastasis.

### TIM3 is upregulated in metastatic clinical samples

TIM3 expression was confirmed to be expressed in tumor cells by co-localization with mammaglobin (MGB1), a marker of breast cancer epithelial cells,^36^ detected by immunofluorescence (IF), and by IHC TIM3 staining of 75 patients samples with primary-metastasis matched tissues from the ConvertHER cohort^37^ (NCT01377363) (Figure 2H). TIM3 positivity in tumor cells was increased in metastasis compared to primary-matched tumors and also the TIM3 tumor cell scoring (Figure 2I). TIM3 was also higher in metastatic samples in stromal tumor-infiltrating lymphocytes (sTILs), intratumoral-infiltrating lymphocytes (iTILs), and a trend in tumor-associated macrophages (TAMs) (Figure S2K). These results confirmed the striking TIM3 expression in tumor cells in breast cancer patient samples of all subtypes and the TIM3 upregulation in metastatic clinical disease.

### TIM3 is associated to EMT-like cells and triggers β-catenin signaling

**Figure 3:**
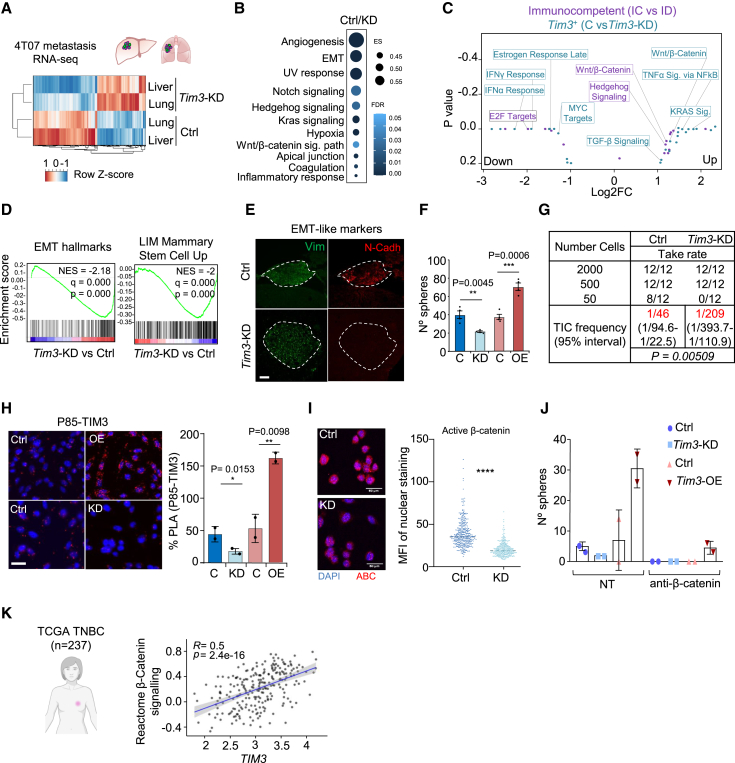
TIM3^+^ MICs display Stemness/EMT-like features and β-catenin activation (A) Unsupervised hierarchical clustering heatmap of the indicated conditions from 4T07 metastases RNA-seq analysis (*n* = 2 independent biological replicates). (B) GSEA from (A) experiment comparing Ctrl and *Tim3*-KD (*n* = 2 independent biological replicates). (C) Gene ontology integration of the metastasis immunoediting RNA-seq from Figure 1A and the *Tim3*-KD metastasis RNA-seq (A). (D) GSEA ranked list Ctrl and *Tim3*-KD 4T07 tumors (lung and liver metastases) interrogated with the indicated EMT-like and stem-like gene signatures. (E) Tissue immunofluorescence representative image: for N-cadherin (red) and vimentin (green) in Ctrl and *Tim3*-KD metastatic livers. Dash line delineates the metastatic tissue. Scale bars, 100 μm. (F) Tumorsphere quantification at day 5 after seeding 500 4T07 cells of indicated conditions (*n* = 3 independent biological replicates). Data represented as mean ± SEM. (G) MFP injection and limiting dilution assay (LDA) of 4T07 Ctrl and *Tim3*-KD cells. Table represents serial dilution injections and tumor take rate. Tumor-initiating cell (TIC) frequency calculated by ELDA software shown in red. *p* value by Pearson’s Chi-squared two-tailed test. (H) Proximity ligation assay showing the interaction (red dots) of P85 and TIM3 in 4T07 Ctrl and *Tim3*-KD cells. Quantification of the interactions per area. Data represented as mean ± SEM. (I) Immunofluorescence of active β-catenin (ABC) in 4T07-Ctrl and *Tim3*-KD cells. Quantification of the nuclear staining of ABC. (J) Tumorsphere quantification of 4T07 Control, *Tim3*-KD, and *Tim3*-overexpression upon 20 μM dose of β-catenin inhibitor. (K) Rho correlation of *TIM3* mRNA levels and the Reactome β-Catenin signaling signature in 237 TNBC patients TCGA. R^2^ and *p* value are shown. Statistical significance; ^∗^*p* < 0.05, ^∗∗^*p* < 0.01, and ^∗∗∗^*p* < 0.001, by two-tailed Student’s t test in (F) and (H); unpaired Student’s t test in (I). Also see Figure S3.

To understand the mechanistic actions of TIM3 in BC metastasis, we performed RNA-seq of *Tim3*-KD and control 4T07 tumor cells isolated from metastasis after 2 weeks of i.c. injection. Transcriptomic profiles of lung and liver metastasis samples clustered according to *Tim3* status and not by colonized organs (Figures 3A and S3A). GSEA revealed EMT, Wnt/β-catenin signaling, and other stemness related pathways enriched in *Tim3*-positive metastatic tumors (Figure 3B). CellMarker^38^ confirmed the enrichment of mesenchymal-like and stem cell-like pathways (Figure S3B). These results are aligned with the biology of MICs^5^^,^^39^ and the metastasis immunoediting experiments (Figure 1E). Wnt/β-catenin signaling was enriched in TIM3^+^ (Ctrl) vs. *Tim3*-KD datasets and in IC vs. ID (Figure 3C). TIM3^+^ (control) vs. *Tim3*-KD cells were enriched in EMT hallmarks and LIM Mammary stem cells^27^ genesets (Figure 3D). In addition, several β-catenin targets were upregulated (Figure S3C), related with β-catenin-mediated immunosuppression and stemness.^40^^,^^41^ Altogether, these observations suggest a pivotal role of TIM3 driving tumor immune-evasive stem cell-like phenotypes.

Tissue immunofluorescence in TIM3^+^ (control) and *Tim3*-KD 4T07 cells metastasis samples showed high levels of N-cadherin, vimentin, and low E-cadherin (Figures 3E and S3D), which are classic EMT markers. *Tim3* levels were found consistently upregulated during EMT induction using public datasets (Figure S3E). In addition, *in vitro* functional assays for stem cell-like properties showed increased tumorsphere formation according to TIM3 levels in 4T07 cells: *Tim3*-OE > control >*Tim3*-KD (Figure 3F). To assess the tumor initiating capacity (TIC) as a typical measure of tumorigenicity and stemness, limiting dilution assay (LDA)-MFP injection of 4T07 cells in immunodeficient mice showed reduction of TIC frequency when *Tim3* was knocked down (Figure 3G). In agreement, gene expression analysis of embryonic stem cell factors involved in breast cancer^42^ were downregulated in *Tim3*-KD cells (Figure S3F). GSEA showed positive correlation of TIM3^+^ cells with Wong CSCs^43^ and Yamashita liver CSCs^44^ genesets (Figure S3G). In addition, GSVA^45^ confirmed the correlation of *TIM3* expression with the CSC_GUPTA signature^46^ in TCGA-BRCA clinical samples (Figure S3H). Overall, these functional and computational analyses confirmed the EMT-like stemness phenotype of TIM3^+^ MICs supporting tumor-initiation capabilities.

TIM3 has a cytoplasmatic domain with tyrosine residues, and it is reported to interact with PI3K in immune cells^31^ and to activate AKT/β-catenin signaling in leukemia cells.^47^ Accordingly, we found β-catenin signaling enriched in our RNA-seq data (Figure 3C). Proximity ligation assay (PLA) of the PI3K subunit P85 and TIM3 showed strong interaction in BC cells (Figure 3H). Using a phospho-kinase array available for human cells, we observed a reduction in phosphorylation of the glycogen synthase kinase 3 α/β (GSK3-α/β) in *TIM3*-KD MDA-MB-231 cells (Figure S3I) and in mouse 4T07 *Tim3*-KD cells detected by western blot (Figure S3J). The dephosphorylated (active) from of GSK3 inhibits β-catenin.^48^ Hence, active β-catenin was reduced in 4T07 *Tim3*-KD cells measured by IF (Figure 3I). Instead, *Tim3*-OE increased nuclear β-catenin in 4T07 cells (Figure S3K). Moreover, β-catenin inhibition demonstrated that β-catenin was required for TIM3-mediated tumorsphere formation (Figure 3J) in agreement with the known functions of β-catenin in EMT and stemness.^41^^,^^47^^,^^49^^,^^50^ Moreover, using clinical data from the TCGA-BRCA TNBC cohort, *TIM3* expression correlated with the β-catenin signaling (Figure 3K). Overall, TIM3^+^ MICs display pro-survival EMT-like stem cell phenotype related to the β-catenin signaling, which is also related to immunosuppressive effects.^40^^,^^41^

### Spatiotemporal analysis of TIM3^+^ tumor cells in micrometastasis

**Figure 4:**
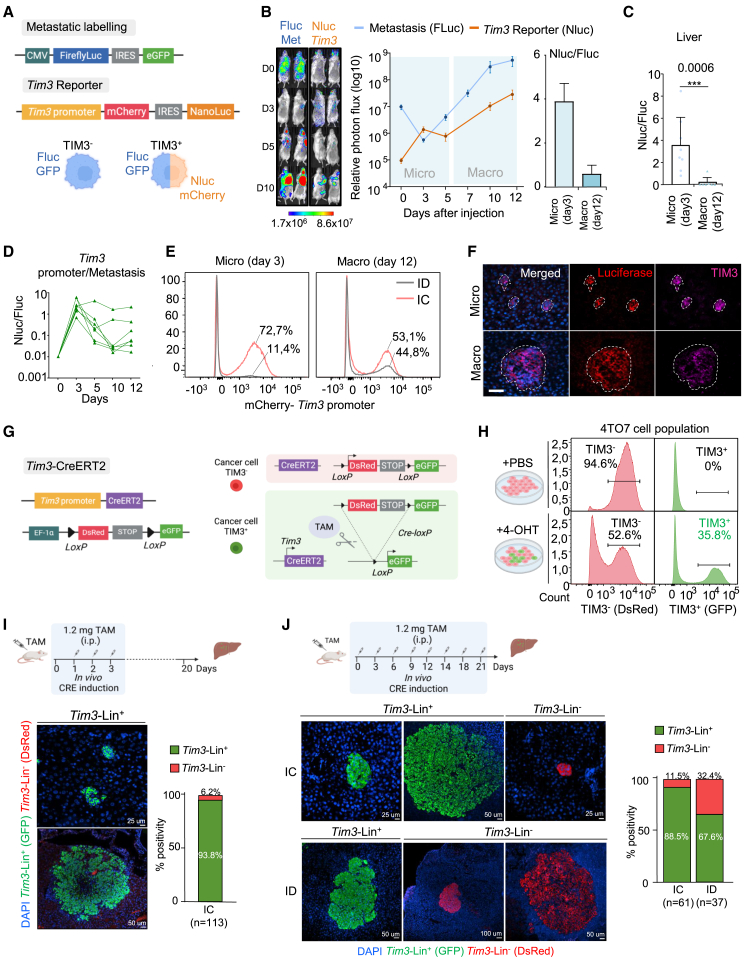
TIM3 spatiotemporal dynamics in metastasis (A) Dual reporter system designed to track bulk tumor metastasis (Firefly luciferase [Fluc]-eGFP) and *Tim3* promoter activity (mCherry-Nanoluciferase [Nluc]). (B) Representative BLI images of Fluc and Nluc signal in IC mice (Balb/c) upon i.c. injection of 4T07 cells experimental metastasis. BLI monitorization of metastatic growth: metastasis curve (blue line) and *Tim3* reporter curve (orange line). Bar plot shows the BLI signal ratio of NLuc/FLuc representing the intensity of the *Tim3* reporter signal versus the overall bulk tumor metastasis at micrometastasis and macrometastasis time points. *n* = 8 mice. (C) BLI ratio of NLuc/FLuc of liver metastasis. *n* = 8 mice. Statistical significance; ^∗^*p* < 0.05, ^∗∗^*p* < 0.01, and ^∗∗∗^*p* < 0.001, by one-tailed Student’s t test. (D) BLI ratio curve of NLuc/FLuc metastasis along days of experiment. Each line represents individual mice. (E) Flow cytometry of 4T07-*Tim3* reporter positivity measured by mCherry intensity of liver metastatic digested tissues from ID and IC at micrometastasis and macrometastasis time points. Representative plot of *n* = 3 individual mice per time point. (F) IF images from liver micrometastasis and macrometastasis. Luciferase (red) and TIM3 (pink) stainings. Dash line delineates the metastatic tissue. Scale bars, 100 μm. (G) Schematic representation of the lineage tracing system introduced in 4T07 cells (see STAR Methods for details). *Tim3*^*-*^ cancer cells have red fluorescence of dsRed. *Tim3*^*+*^ cancer cells have green fluorescence of eGFP. (H) *In vitro* lineage tracing test of *Tim3*^*+*^ and *Tim3*^*-*^ 4T07 cells. Induction by 4-hydroxy-tamoxifen (4-OHT) O/N at 1 μM. Flow cytometry plots represent dsRed and GFP positive events in no induced cells (top) and 4-OHT induced cells (bottom). (I) Metastasis lineage tracing using 4T07 cells after intracardiac injection in IC mice. Short TAM induction during the first 3 days of metastatic seeding (see STAR Methods). Bar plots quantifications of all lesions (113) present in livers of 6 independent experiments. Representative immunofluorescence images of liver sections showing TIM3^+^ (green) and TIM3^-^ (red) metastasis in (I) and (J). (J) Long-term TAM induction during 21 days after intracardiac injection of 4T07 cells in IC and ID mice. Bar plots quantifications of all lesions (61 IC and 37 ID) present in livers of 4 independent experiments. Also see Figure S4.

In order to capture the initial events of metastasis seeding of TIM3^+^ cells in our models, we monitored 4T07-FLuc-GFP cells by BLI imaging at 0, 6 h, and 3 days after i.c. systemic delivery, showing tumor cells distributed in different organs after 6 h (Figures S4A and S4B). Microscopy of tissue sections demonstrated the presence of DTCs within the liver tissue parenchyma already 6 h after administration (Figure S4C). After 3 days, there was a drop of BLI signal in immunocompetent mice (Figure S4A–S4D), indicating that many DTCs perished at the early days of metastatic tissue seeding, in agreement with previous knowledge.^51^ Next, to study the temporal dynamics of TIM3 during the early and late stages of metastasis, we designed a dual-reporter system to follow *Tim3* levels *in vivo* (Figure 4A). This system reports *Tim3* promoter activity by mCherry fluorescence and nano-luciferase (Nluc) (high sensitivity) allowing monitoring *Tim3* expression by BLI (Figure 4A). We validated the precision of the reporter using anti-TIM3 flow cytometry detection and mCherry positivity by flow cytometry in 4T07 cells (Figure S4E).

Using this dual-reporter system (constitutive Fluc and TIM3-Nluc-reporter), we performed experimental metastasis assays with 4T07, 66cl4, and EpRas cells via intracardiac delivery. BLI measurements of metastasis and *Tim3* activity were assessed by luciferin (Fluc signal) and coelenterazine administration (Nluc signal), respectively, six hours apart to avoid BLI signal overlap. This dual system showed how the whole tumor cell burden decreased suggesting massive cell death at the moment of organ seeding, while TIM3^+^ cells were positively selected and growing proving their survival advantage at initials days (Figures 4B and S4F). Using the Nluc/Fluc signal ratio, we revealed a remarkable increase of *Tim3* levels specific of the early days (0–4 days) of metastasis seeding of 4T07 cells (Figures 4B–4D). After day 5, tumor cell burden started to increase, and *Tim3*^+^ cells showed a similar increase rate as the rest of the bulk tumor cells (Figure 4B). Consistently, similar *Tim3* survival dynamics were observed using other metastasis models (Figure S4G). These results suggested that TIM3-negative cells mostly died during the initial days of seeding and TIM3^+^ cells were positively selected by surviving this phase of metastasis. BLI localized measurements captured the same *Tim3* dynamics in liver, lung, and brain 4T07 micrometastasis when comparing metastasis at day 3 and 12 (Figures 4C and S4H), being the liver the organ with the highest ratio of TIM3^+^ levels in accordance to our results (Figures 2B and S1G). Remarkably, tumor cell attrition was not occurring in ID mice and accordingly TIM3 levels did not increase, indicating that *Tim3*^+^ cells were not critical to lead metastasis in ID mice (Figures 4D and S4I). These results suggest a crucial role of TIM3-mediated survival by immune-evasion during the metastatic seeding and micrometastasis progression to macrometastasis.

These results were further validated by harvesting 4T07 metastatic livers at day 3 and 12 after i.c. injection (Figures 4E, 4F, and S4J). Flow cytometry measurements showed that most of tumor cells were TIM3^+^ during the early days of micrometastasis in IC hosts but not in ID hosts (Figure 4E). Accordingly, tissue immunofluorescence confirmed that all tumor cells were TIM3^+^ at the moment of micrometastasis in the liver (Figure 4F). Instead, the percentage of TIM3^+^ cells decreased after 12 days in macrometastasis compared to the micrometastasis stage, suggesting cellular plasticity and differentiation of TIM3^+^ MICs into TIM3^-^ tumors cells (Figures 4E and 4F).

To understand the cellular plasticity of TIM3 expression and the potential origin of the metastasis related to TIM3-derived cells, we engineered a *Tim3* lineage tracing system using the CreERT2 recombinase. We generated stable random integration *LoxP-DsRed-STOP-LoxP-eGFP* 4T07 cells with the CreERT2 expression driven by *Tim3* promoter, activated after tamoxifen induction (Figure 4G). The system was tested *in vitro* and *in vivo* showing that it was precisely tamoxifen activated (Figure 4H), and no leakiness was detected in the absence of tamoxifen *in vivo* (Figure S4K). Of note, the *in vitro* test at day 0 showed similar percentage of TIM3-positive 4T07 cells as measured by flow cytometry, thus validating the specificity of the system (Figures 1H and S1E). Next, we performed i.c. experimental metastasis with 4T07 cells and activated the Cre with tamoxifen treatment right before injection of the cells and during the initial 3 days after injection, followed by treatment withdrawal. After 20 days of experiment, liver tissues were harvested and studied by microscopy to detect the fate-labeling of the metastases. The results showed >93% of GFP-positive cases, indicating that most of the metastases originated from the *Tim3*-lineage, with a rare minority coming from the *Tim3*-negative lineage (Figure 4I). Longer activation tamoxifen regimes (20 days) did not show additional % of metastases originated from the *Tim3*-lineage compared to 3-day induction (Figure 4J). This was in agreement with the peak of expression of TIM3 during the initial days of micrometastasis (Figures 4D–4F). In immunocompromised mice, the *Tim3*-lineage did not show to be as prevalent, with only 67.6% of GFP *Tim3*-lineage metastases (Figure 4J). This represents a modest increment considering the starting point of positivity in 4T07 cells *in vitro* was 36% at the moment of injection (Figure 4H). Therefore, the lineage tracing supported that most of metastases were derived from TIM3^+^ lineage MICs, and immune pressure executed a positive selection specific of the moment of micrometastasis. Yet, in permissive immunodeficient environments, TIM3^+^ cells participate in metastasis associated to intrinsic MICs properties, including EMT and stemness. Overall, TIM3^+^ MICs are specifically advantageous during micrometastasis, through extrinsic immune-evasive and intrinsic EMT/stem cell like properties. These findings are aligned with the EMT/stemness metastatic seeding and posterior reversion to form macrometastasis.^39^^,^^50^^,^^52^^,^^53^^,^^54^^,^^55^

### TIM3^+^ MICs reconfigure the immunosuppressive immune landscape of micrometastasis

**Figure 5:**
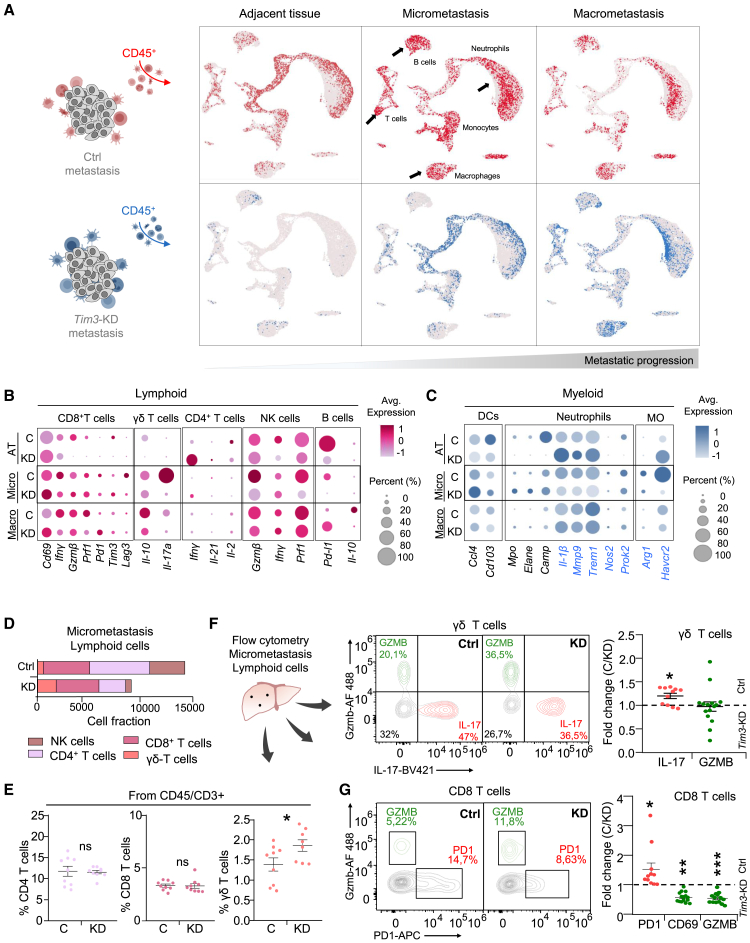
TIM3^+^ MICs induce an immunosuppressive environment during micrometastasis (A) Single cell RNA-seq uniform manifold approximation and projection (UMAP) of CD45^+^ immune cells isolated from liver metastasis after 4T07 Ctrl and *Tim3*-KD i.c. injection. UMAP representation of: Ctrl (red) and *Tim3*-KD (blue) in healthy liver adjacent tissue (AT), liver micrometastasis and liver macrometastasis. (B) scRNA-seq bubble plot showing average expression (color) and percentage expression (size) of different genes defining lymphoid annotated cell compartments across metastatic conditions. Average expression legend is shared among populations. Percentage expression is relative within each immune population type. (C) Idem B for the myeloid compartment. (D) scRNA-seq cell fraction of the lymphoid cell compartment in micrometastatic samples from 4T07 Ctrl and *Tim3*-KD conditions. (E) Flow cytometry analysis of CD4^+^ T cells, CD8^+^ T cells and γδ T cells from liver micrometastasis from 4T07-Ctrl and 4T07-*Tim3*-KD. Data represents mean ± SEM, *n* = 11 independent biological replicates in (E), (F), and (G). (F) Flow cytometry of cytotoxic γδ T cells (GZMB^+^) and immunosuppressive (IL17^+^) γδ T cells. Dot plot represents fold change of IL-17 and GZMB γδ T cell populations in TIM3+ (Ctrl) vs. TIM3- (*Tim3*-KD) micrometastasis. (G) Flow cytometry of cytotoxic CD8^+^ T cells (GZMB^+^, CD69^+^) and exhausted T cells (PD1^+^) populations. Dot plot represents fold change of PD1, CD69, and GZMB positivity in TIM3^+^ (Ctrl) vs. TIM3^-^ (*Tim3*-KD) micrometastasis. Statistical significance respect to the Ctrl; ^∗^*p* < 0.05, ^∗∗^*p* < 0.01, and ^∗∗∗^*p* < 0.001, by unpaired Student’s t test in (E), (F), and (G). Also see Figures S5, S6, and Table S2.

In order to understand the TIM3^+^ tumor cell-mediated immune-evasive effects during micrometastasis (in 4T07 Ctrl and *Tim3*-KD conditions), we isolated CD45^+^ immune cells as described^56^ and we performed single-cell RNA-seq from micro- and macro-metastatic liver and adjacent tissue (AT) (Figure 5A and S5A–S5C). Micrometastasis was taken by *ex vivo* BLI signals not detected by the human eye (Figure S5B). The Leiden algorithm generated 27 clusters of immune populations annotated according to differential gene expression (Figures S5D–S5F). Our analysis showed a good representation of lymphoid and myeloid lineages and subset populations validating the quality of immune cell isolation.

Across our conditions of AT, micrometastasis and macrometastasis, we observed dynamic changes of the different immune cell fractions, and most relevant changes occurred in micrometastasis (Figures 5A and S5F). The biggest differences of immune populations between control (TIM3^+^) and *Tim3*-KD samples were also observed in micrometastasis. In general, there was a gain of immunosuppressive immune cells in TIM3^+^ micrometastasis compared to *Tim3*-KD (Figures 5B and 5C). In detail, comparing control (TIM3^+^) vs. *Tim3*-KD, we identified an increase of immunosuppressive populations, in particular of *Il-17* γδ T cells in micrometastasis (Figure 5B) and myeloid immunosuppressive populations,^57^ such as *Il-1β*^*+*^,^58^ *Mmp9*, *Trem1*, *Nos2*, *Prok2* neutrophils, and *Arg*1^+^, *Tim3* monocytes^59^ (Figure 5C). B cell compartments were also affected and lost in *Tim3*-KD micrometastasis (Figure 5B). In contrast, anti-tumoral immune populations were reduced in TIM3^+^ (Ctrl) micrometastasis compared to the *Tim3*-KD, such as *CD69*^+^ and *Gzmb* effector CD8^+^ T cells, dendritic cells (DCs), or Elane^+^ Camp, Mpo neutrophils^60^^,^^61^^,^^62^ (Figures 5B and 5C). To further explore the potential immune-to-immune cell interactions based on the scRNA-seq data, we used the LIANA Tensor cell-cell algorithm.^63^ The interacting factors of receiving and sender cells were more enriched in micrometastases than macrometastases (Figure S5G). *In silico*, the LIANA factor-4 included a core of interactions of lymphocytes and γδ T cells with immunosuppressive myeloid cells, which was reduced in the *Tim3*-KD micrometastasis (Figure S5H and Table S2). Overall, the single-cell transcriptomic analysis was informative in understanding the immune landscape of micrometastasis, which revealed an early induction of an immunosuppressive immune microenvironment in liver micrometastasis mediated by TIM3^+^ MICs.

Next, spectral flow cytometry analysis of liver micrometastases (Figures S6A and S6B) validated the distribution and percentage of the different cell types of the lymphoid lineage and the myeloid lineage among TIM3^+^ (Ctrl) and *Tim3*-KD (Figures S6C–S6G) observed in the scRNA-seq results (Figures 5D and S6F). We confirmed the increase of immunosuppressive IL-17 γδ T cells in Ctrl (TIM3^+^) liver micrometastasis compared to *Tim3*-KD micrometastasis (Figure 5E). Cytotoxic GZMB γδ T cells did not show consistent changes among these conditions. Moreover, we also validated the reduction of activated (CD69 and GZMB) effector CD8^+^ T cells in TIM3^+^ (Ctrl) micrometastasis (Figure 5F) supporting the scRNA-seq analysis. Overall, these results suggest that TIM3^+^ MICs orchestrate a permissive immunosuppressive liver microenvironment specific of micrometastasis, with increased IL-17 γδ T cells and low cytotoxic CD8^+^ T cells.

### γδ T cells are important players during liver micrometastasis

**Figure 6:**
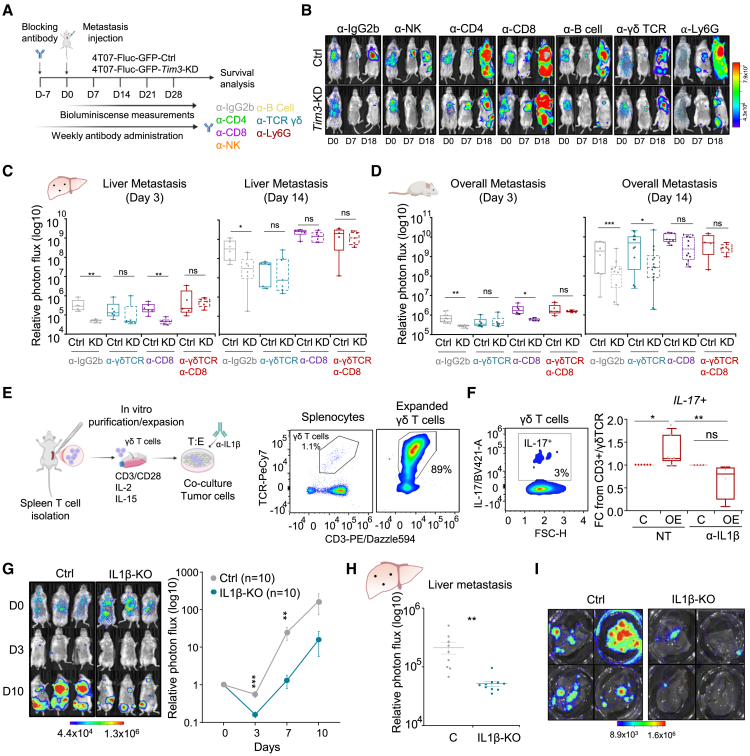
Functional metastasis assessment of γδ T cells, CD8 T cells, and IL-1β in TIM3-mediated immunosuppression (A) Blocking antibody scheme; intraperitoneal administration and doses indicated. Seven days before 4T07 tumor cell i.c. injection and, weekly reminder of 250 μg of antibody during the experiment. (B) Representative BLI images of 4T07 whole body metastasis for the indicated immune blocking conditions. (C) Boxplot quantification of liver metastasis at micro (day 3) and macro (day 14) time points upon IgG2b, CD8, γδ TCR, and double (CD8, γδ TCR) cell depletion. Each dot represents independent mice. (D) Boxplot quantification of whole-body metastasis at micro (day 3) and macro (day 14) time points upon IgG2b, CD8, γδ TCR, and double (CD8, γδ TCR) cell depletion. Each dot represents independent mice. (E) *In vitro* proliferation and co-culture of γδ T cells with 4T07 tumor cells and flow cytometry. See STAR Methods for details. (F) Flow cytometry quantification of IL-17 γδ T cell levels after co-culture in non-treated (NT) conditions and upon anti-IL1β treatment. Data represents mean ± SEM, *n* = 6 (NT) and *n* = 4 (anti-IL1β) independent biological replicates. (G) Whole-body metastasis assays after systemic delivery of 4T07-IL-1β*-*KO cells compared to 4T07 TIM3^+^ (Ctrl) cells. Representative BLI images of metastasis at the indicated conditions. BLI metastasis growth curves. Data represents mean ± SEM, *n* = 10 independent mice. (H) Liver metastatic lesions at day 3 of metastatic seeding of 4T07-IL-1β-KO cells compared to 4T07 TIM3^+^ (Ctrl) cells. (I) Representative BLI images of *ex vivo* metastatic livers at day 14 after i.c. injection of 4T07-Tim3^+^ (Ctrl) and 4T07-IL-1β-KO cells. Statistical significance respect to the Ctrl; ^∗^*p* < 0.05, ^∗∗^*p* < 0.01, and ^∗∗∗^*p* < 0.001, by two-tailed Student’s t test in (C), (D), (F), and (G); unpaired Student’s t test in (H). Also see Figure S7.

To functionally validate relevant immune cell populations mediating TIM3^+^-MIC effects in the transition from micro-to-macro metastasis, we used neutralizing antibodies to deplete or block specific immune cell populations in IC Balb/c mice, focusing in the most relevant observed in the scRNA-seq data: γδ T cells, CD8^+^ T cells, and also CD4^+^ T cells, NK cells, B cells, and neutrophils (Figures 6A and S7A). We performed metastasis rescue experiments comparing 4T07 TIM3^*+*^ (Ctrl) and the *Tim3*-KD condition subjected to blocking antibody treatments specific of the immune populations mentioned, and following the metastatic growth (liver and whole-body metastasis) and mice survival (Figures 6A, 6B, and S7B).

γδ T cell neutralization decreased liver metastasis of TIM3^+^ control cells to a similar level of *Tim3*-KD cells suggesting that, without immunosuppressive γδ T cells, TIM3^+^ cells lose their selective advantage during liver micrometastasis to macrometastasis validating their implication in liver micrometastasis and overall metastasis (Figures 6C and 6D). These effects persisted at later time points liver metastasis, but not in other organs (Figures 6B and 6C) nor survival effects (Figure S7B), suggesting a more prominent role of γδ T cells in liver metastasis than other organs. Regarding CD8^+^ T cells, their blocking rescued the liver and whole-body metastatic ability of *Tim3*-KD cells reaching same capacity as TIM3^+^ cells at later time points (day 14) (Figures 6C and 6D). Moreover, this specific depletion resulted in poor mice survival of the *Tim3*-KD condition similar to the control-TIM3^+^ cells (Figure S7B). Considering the importance of the interplay between both populations during the dynamics of micro-to-macro metastasis, we performed a double neutralization of γδ T cells and CD8^+^ T cells, which resulted in a complete loss of the *Tim3*-KD differential effect in micrometastasis and macrometastasis time points, both in the liver (Figure 6C) and overall metastasis (Figure 6D), underscoring the relevance of both cell types in the process of micro-to-macro-metastasis. These results were aligned with the results obtained in NSG mice (Figures 2A and S2B). In contrast, when evaluating the depletion of CD4^+^ T cells, B cells, neutrophils, and NK cells, we did not observe conclusive effects as none of them showed a clear rescue in metastatic growth of *Tim3*-KD cells (Figures S7C and S7D) neither reduced the extended survival of *Tim3*-KD compared to control-Tim3^+^ conditions (Figure S7B). Overall, these results functionally validated the implications of γδ T cells mediating the TIM3^+^ licensing of micrometastasis, especially in the liver, and CD8^+^ T cells as key players preventing metastasis of TIM3^-^ cells.

The induction of IL-17 γδ T cells has been reported to be mediated by IL-1β from immune cells.^64^ Moreover, β-catenin signaling induces IL-1β,^65^ which is confirmed in our models with high IL-1β expression in *Tim3*-OE cells and downregulated IL-1β in *Tim3*-KD 4T07 cells (Figure S7E). Hence, we performed co-culture of *Tim3*-OE tumor cells with isolated γδ T cells *in vitro* as described^66^^,^^67^ (Figure 6E). The co-culture of *Tim3*-OE cells modestly increased IL-17 levels in γδ T cells, although relevant in these *in vitro* settings. Importantly, blocking IL-1β prevented and reduced the levels of IL-17 γδ T cells (Figures 6F). Due to the limitations of working with γδ T cells *in vitro*,^68^ we explored the impact of IL-1β-KO (knock-out) in TIM3^+^ 4T07 cells *in vivo* (Figure S7F). IL-1β-KO TIM3^+^ cells drastically reduced their metastatic seeding in different organs (Figure 6G), specifically in the liver (Figure 6H), and impaired posterior development of liver metastasis (Figure 6I). Overall, these *in vitro* and *in vivo* functional assays revealed mechanistic insights validating the TIM3^+^/β-catenin/IL-1β axis in promoting IL-17 γδ T licensing micrometastasis.

### TIM3-expressing tumor cells independently predict poor breast cancer patient outcome

**Figure 7:**
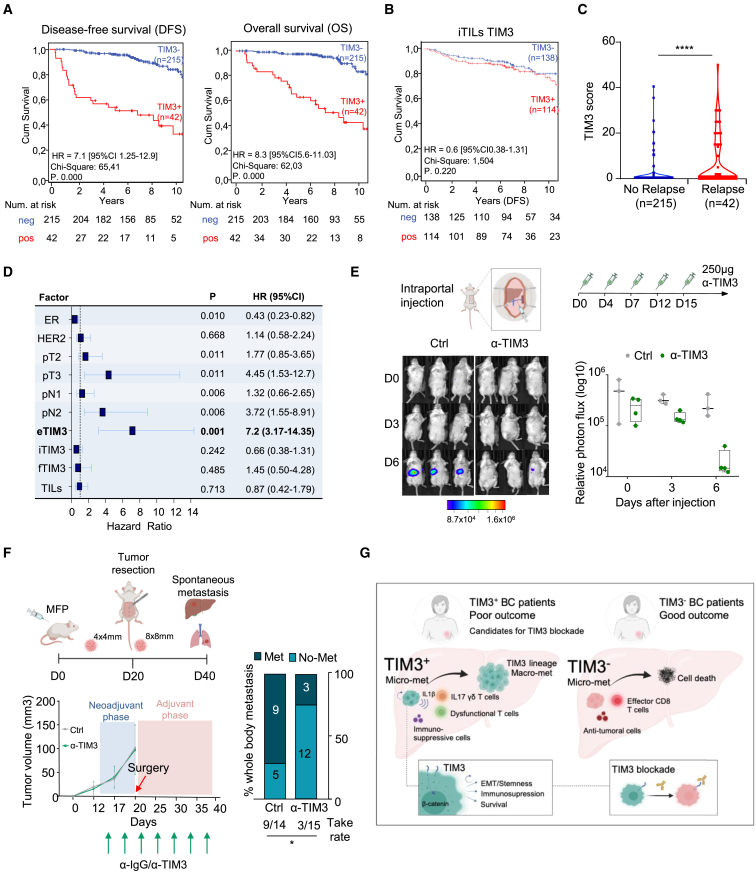
Clinical and preclinical evaluation of TIM3 blockade for breast cancer metastasis (A) Disease free survival (DFS) and overall survival (OS) Kaplan-Meier curves of IHC epithelial-tumor TIM3^+^ and TIM3^-^ breast cancer primary tumor samples. Data obtained from Tissue microarrays (TMAs) with 257 breast cancer primary tumors from all subtypes. Statistical significance calculated by Log Rank (Mantel-Cox) for Chi-square and *p* value. (B) DFS Kaplan-Meier curves of IHC intratumoral tumor infiltrating lymphocytes (TILs) TIM3^+^ and TIM3^-^ breast cancer primary tumor samples. TMAs with 252 breast cancer primary tumors from all subtypes. Statistical significance calculated by Log Rank (Mantel-Cox) for Chi-square and *p* value. (C) Violin plot of TIM3 score in patients stratified by relapse (*n* = 42) and non-relapse (*n* = 215) from breast cancer primary tumor samples. ^∗^*p* < 0.05, ^∗∗^*p* < 0.01, and ^∗∗∗^*p* < 0.001, by two-tailed Student’s t test. (D) Multivariate Cox regression analysis of TIM3 IHC from previous samples including *p* value and hazard ratio (HR) with confidence interval. Factor names: TIM3 in tumor epithelial cells (eTIM3), intratumoral TILs (iTIM3), and fibroblasts (fTIM3). Estrogen-receptor positivity (ER), HER2 positivity (HER2), patient stage-II BC (pT2), patient stage-III BC (pT3), patient lymph node-1 (pN1), patient lymph node-2 (pN2), and tumor infiltrating lymphocytes (TILs). (E) Intraportal 4T07 cell injection and anti-TIM3 treatment. Representative images of liver metastasis of anti-IgG2a and anti-TIM3 conditions. Boxplots representing liver metastatic growth by BLI measurements during early time points of the experiment. Each dot represents an individual mouse. (F) Spontaneous metastasis assay using 4T1 MFP injection in Balb/c mice. At 4 × 4 mm size, anti-Tim3 treatment starts. On the left, the graph represents the primary tumor volume, and the scheme of neoadjuvant/adjuvant treatment regime of TIM3-blockade therapy (250 μg). On the right, bar plot quantification of spontaneous metastatic incidence at day 40 and take rate of metastasis incidence. Met (metastasis detection) or No-Met (no metastasis detection). *n* = 14 and *n* = 15 mice per condition. Statistical significance; ^∗^*p* < 0.05, ^∗∗^*p* < 0.01, and ^∗∗∗^*p* < 0.001, by Chi-square test. (G) Graphical abstract of TIM3^+^ tumor cells from early seeding to macrometastasis in the liver. Also see Figure S8.

To evaluate the risk of metastasis associated to TIM3 expression in tumor cells, we used 257 primary tumor samples from breast cancer patients including all subtypes and I-III disease stages (Figure S8A). IHC revealed that TIM3 expression in tumor cells was strongly associated to worse prognosis for disease-free survival (DFS) and overall survival (OS) in all subtypes (Figures 7A and S8B). Remarkably, TIM3 score in iTILs did not predict patient outcome (Figure 7B). The TIM3 expression score in tumor cells was significantly enriched in the relapsing patients (Figure 7C), which posits TIM3 as predictive biomarker determined by the univariate ROC analysis (Figure S8C). Multivariate Cox regression analysis showed how TIM3^+^ in tumor cells (eTIM3), but not in other compartments (TILs or fibroblasts), was an independent prognostic factor for DFS with a hazard ratio 7.2 CI 95% (Figure 7D). Among subtypes, TNBC concur with the worst prognosis of TIM3^+^ patients (Figure S8B) and across stages the worse prognosis was at high-risk stage-III (Figure S8D). These findings support the potential use of eTIM3 as biomarker for the stratification of high-risk relapse BC patients.

### Anti-TIM3 therapy to thwart immune-evasive MICs

TIM3 blockade agents are already being used in clinical trials as ICIs. However, here, we used TIM3 blockade therapy to target MICs during micrometastasis. First, to study TIM3 blockade in liver metastasis seeding, we performed intraportal vein injection for liver metastasis. In this experimental model, anti-TIM3 (Bio X cell) therapy reduced metastatic seeding and survival at the early time points (days) of metastatic initiation in the liver (Figure 7E). Second, in experimental metastasis assays after i.c. 4T07 injection, mice were treated every 4 days with 250 μg/mL of anti-TIM3 via i.p. injection. Treated mice showed reduced overall metastasis compared to non-treated animals. More than 55% of the treated mice responded to anti-TIM3 therapy (Figure S8E). Of note, anti-TIM3 therapy phenocopied the metastatic decrease of *Tim3*-KD cells, and the administration of anti-TIM3 plus *Tim3*-KD condition did not present significant additional suppressive effects (Figure S8F), suggesting that the effects of anti-TIM3 are mostly mediated by targeting TIM3 in tumor cells.

Next, with the aim to mimic a clinical therapeutic scenario, we assessed TIM3 blockade therapy in a neoadjuvant/adjuvant (NA/A) setting to reduce and prevent breast cancer metastasis. For this purpose, we used spontaneous metastasis assays with MFP of 4T1 cells followed by tumor resection to allow posterior metastasis development. The neoadjuvant anti-TIM3 phase started when tumors reached 4 × 4 mm, and the adjuvant phase continued after tumor resection when tumors reached 8 × 8 mm. In this setting, anti-TIM3 therapy did not show difference in primary tumor growth, which phenocopies the lack of effect of *Tim3*-KD in primary tumor growth (Figure 2F). Remarkably, as occurring with the *Tim3*-KD (Figure 2A), the NA/A TIM3 blockade reduced metastasis and prevented 80% of lung and liver metastasis incidence at day 40 (Figures 7F and S8G, 8H), suggesting a promising MIC-targeting therapy to prevent metastasis. Thus, our results in clinical samples and preclinical models suggest the initiation of clinical studies of (neo)adjuvant TIM3 blockade for TIM3^+^ stage-II/III high-risk breast cancer patients to halt subclinical metastasis.

## Discussion

In this study, by investigating the metastatic colonization dynamics influenced by the immune pressure in distant organs, we discovered a mechanism specific of micrometastasis immune evasion. We demonstrate that TIM3 is critical for the seeding and survival of micrometastasis, when stemness and immune-evasion are required to overcome the challenges of the distant tissues (Figure 7G). A main observation of our study is that the tumor biology and the immune microenvironment of micrometastasis are governed by distinct mechanisms compared to macrometastases, indicating a necessity to consider appropriate (neo)adjuvant therapeutic strategies, especially immune-based therapies at this disease stage.

Our results provide evidence for the existence of phenotypic metastasis immunoediting in distant organs. Experimental metastasis assays revealed how the immune selective pressure selects MIC-like aggressive tumor phenotypes escaping immunity associated with stemness and EMT traits. This is consistent with the phenotypes found in clinical breast cancer datasets from metastatic samples.^29^^,^^30^ Additionally, stem cell-like phenotypes have been shown to have immune evasive properties, from embryonic stem cells, adult stem cells, to cancer stem cells,^7^^,^^9^^,^^69^^,^^70^ including metastatic latency and regenerative programs in metastasis.^6^^,^^71^^,^^72^ Altogether suggests that stem cell programs are intrinsically linked to immune-evasion from development to malignant metastasis.

Distant organ seeding is a metastasis bottleneck that selects for the fittest cells. Our dynamic *Tim3* reporter and *Tim3*-lineage tracing tools revealed the highest TIM3 levels during micrometastasis and underscored the essential role of TIM3 in supporting the survival of tumor cells in this vulnerable stage, thus originating metastasis. This is a result of TIM3-mediated immunosuppression operating in micrometastasis and further supported by tumor-intrinsic stem cell properties, as suggested by the moderate increase of *Tim3*^+^-lineage cells upon induction in immunodeficient mice, and the TIC ability of TIM3^+^ cells in LDA *in vivo* assays. In macrometastasis, TIM3 exhibits reversibility, although still maintaining higher levels compared to primary tumors as shown in our models and clinical data. These results suggest that TIM3^+^ cells are MICs, and the reversion is in consonance with the MIC principles of cellular plasticity, differentiation, and EMT reversion in overt macrometastasis.^5^^,^^39^^,^^73^^,^^74^

The unexpected expression of TIM3 in breast tumor cells found in this study is in consonance with other studies showing expression of TIM3 in malignant cells in diffuse intrinsic pontine glioma (DIPG),^21^ although the TIM3 blockade effects were mainly occurring in immune-TIM3^+^ microglia and macrophages causing major proinflammatory effects. TIM3 has also been reported to promote proliferation of myeloid leukemia stem cells,^20^^,^^75^ supporting a pro-tumoral role. In breast cancer, *in vitro* studies showed TIM3 expression in cancer cells that led to apoptosis inhibition, proliferation, invasion,^19^ and also T cell inhibition *in vitro* through galectin-9 secretion.^76^ In this context, our results provide important conceptual advance of the role TIM3 in tumor cells in breast cancer metastasis immunity, specifically of MICs at micrometastasis. Importantly, our BC clinical data and preclinical data suggest that TIM3 plays a more relevant role in metastatic tumor cells rather than in immune cells, supporting the use of TIM3 blockade to target tumor cells to prevent metastatic relapse.

We showcase functions of TIM3 associated with EMT-like, stemness phenotypes, and immune-suppression. This includes the enrichment of Wnt, Hedgehog, and Notch pathways in TIM3^+^ cells, which are key EMT and stemness signaling pathways.^41^^,^^77^^,^^78^^,^^79^ These findings are in agreement with the current understanding of plastic EMT hybrid states considered “the seeds” of distant organs and leading aggressiveness.^39^^,^^80^ Mechanistically, we show TIM3/β-catenin signaling in our transcriptomic analyses and validations, in consonance with the TIM3 activation of AKT/β-catenin signaling in myeloid leukemia cells,^20^^,^^47^ and interaction with P85 (PI3KR1),^15^ which is known to inactivate the β-catenin inhibitor GSK3-β.^81^ Therefore, the TIM3/β-catenin axis has previous mechanistic evidence in hematopoietic cells. Importantly, β-catenin is a central player not only in EMT induction and stemness^82^ but also in tumor immunosuppression,^9^^,^^83^^,^^84^ aligned with the immune-evasive stem cell phenotype of TIM3^+^ cells and pro-survival signaling. We identified β-catenin targets upregulated in TIM3^+^ cells, notably IL-1β as a TIM3-mediated inducer of IL-17 in γδ T cells that are reported to have an immunosuppressive role in breast cancer metastasis.^64^^,^^85^ This is in agreement of our results *in vivo* showing that the blocking γδ T cells lose the TIM3 pro-metastatic effect during micrometastasis where TIM3 mediates increased IL-17 γδ T cells.^64^^,^^86^ Our conclusions propose that the TIM3/β-catenin/IL-1β axis is a cornerstone of the micrometastasis immunity.

We focused the study in liver metastasis since TIM3 showed a more prominent role in liver micrometastasis and metastatic outcome. Moreover, liver is a tolerogenic organ and the least responsive to current ICIs, therefore new immune-based therapies are a clinical need. Functional metastasis assays showed a leading effect mediated by γδ T cells, as their neutralization reduced liver micrometastasis to a similar level as *Tim3*-KD cells, suggesting no differential advantageous function of TIM3 in micrometastasis when γδ T cells are absent. Moreover, our results showed the relevant interplay of γδ T cells with CD8^+^ T cells in the dynamics of micro-to-macro metastasis, which mechanistically aligns with the fact that pro-tumoral γδ T cells can suppress CD8^+^ T cells.^87^^,^^88^ Therefore, we unveil a role of γδ T cell specific of micrometastasis.

We have found TIM3 expression as poor prognostic factor only when expressed in tumor cells for the different BC subtypes (TNBC, HER2, and HR^+^ subtypes), and not in TILs. Previous reports show confounding predictive value of TIM3, when TIM3 analysis was assessed only in TILs or in bulk tumor cells including stroma.^89^^,^^90^^,^^91^ Hence, our findings convey that TIM3 should be distinctively evaluated in the tumor compartment for clinical assessment. On the therapeutic side, TIM3 blockade has shown tolerability in clinical assays, without additional toxicity to anti-PD-1/PD-L1 therapies. Our preclinical neoadjuvant-adjuvant TIM3 blockade strategy suggests the potential to eradicate minimal residual disease in high-risk patients, thereby preventing later metastatic outbreaks, an important challenge and unmet need in clinical oncology. This underscores the importance of spatiotemporal understanding of the disease for effective therapeutic intervention against micrometastases or residual disease. Altogether, these findings support the initiation of clinical trials targeting TIM3 to block micrometastasis in patients at high-risk of relapse and poor outcome (eTIM3^+^ stage-II/III patients).

### Limitations of the study

A limitation of preclinical models in immuno-oncology research is the inability to use human cells *in vivo* without compromising immune system integrity. Hence, we used syngeneic mouse breast cancer models to study metastasis while preserving physiological immunity essential to our investigation. Additionally, fluorescent-labeling systems introduce immunogenicity; however, we showed that the TIM3-mediated phenotype was also maintained in unlabeled tumor cells. Our study focuses on immune escape mechanisms independent of neoantigen identity. We employed five distinct murine BC models, including EpRas cells, which are not derived under immune pressure and used in metastasis studies.^22^^,^^23^^,^^24^^,^^25^ We also used established murine metastasis models: 4T1, 4T07, and 66cl4 cells (Balb/c origin), and AT3 cells (C57BL/6 origin). We mostly used 4T07 cells, which offer a clear temporal window for metastasis after systemic delivery, and 4T1 cells, for spontaneous metastasis assays. Both 4T07 and 4T1 are TNBC models with *Trp53* hot-spot mutations,^92^ mimicking the clinical features of human TNBC, which is the subtype with the highest clinical impact of TIM3.

## Resource availability

### Lead contact

Requests for further information should be directed to the lead contact, Toni Celià-Terrassa (acelia@researchmar.net).

### Materials availability

Unique/stable materials generated in this study are available upon request.

### Data and code availability

Raw transcriptomic data have been deposited in Gene Expression Omnibus (GEO) database and are available under accession no.; GEO: GSE260480 (4T07-Ctrl and *Tim3*-KD from lung and liver metastasis), GEO: GSE260481 (EpRas metastasis from different hosts), and GEO: GSE260482 (single cell-RNA sequencing of CD45^+^ cells from 4T07 liver metastasis). The study did not generate new code.

## Acknowledgments

This work was supported by the AECC LAB grant (LABAE19007CELI), FERO foundation (FERO-MANGO, ref PFERO2020.2), Chiara Giorgetti 2021 Asociación Cáncer de Mama Metastásico, the Worldwide Cancer Research charity (grant 20-0156), Generalitat de Catalunya (SGR-22 00037), LaCaixa foundation (HR23-00392), and Instituto de Salud Carlos III-FSE (PI21/00020; CPII22/00001) to T.C.-T. This work was also supported by ISCIII (CIBERONC CB16/12/00241, PI21/00002), AGAUR (2021 SGR 00776), and FEDER to J.A. Also, Postdoctoral AECC 2023 (POSTD234709BLAS) to S.B.B. Also supported by the Spanish Ministry of Economy and Competitiveness (MINECO) with ERDF, ISCIII (AES Program, grant PI21/00142; CIBERONC; Biobank PT23/00114) to F.R. We thank the CRG/UPF flow cytometry assistance. We thank animal facility assistance. Cartoons created with BioRender.

## Author contributions

Conceptualization C.R., I.S., and T.C.-T.; Conception, T.C.-T.; methodological and experimental lead, C.R. with the help of I.S., P.T., S.A., M.S.-F., S.B.-B., J.A.P., M.D., A.C.-M., and I.P.-N.; computational analysis, A.A., P.T., Y.G., and H.B.; tissue histology, S.P.B.; TIM3 IHC analysis, F.R., GEICAM, and J.I.C.; A.G.-Z., E.M.d.D., and B.B. provided the ConvertHER samples; T.M., S.S., J.A., F.R., B.B., and L.C. provided TMA of human samples and data; scientific analytical discussion M.C-A., A.B., J.A., and GEICAM; T.C.-T. and C.R. wrote the manuscript. All authors discussed the results.

## Declaration of interests

L.C. receives personal fees from Roche, MSD, AstraZeneca, Diaceutics; non-financial support Roche, MSD, AstraZeneca, Phillips. F.R. has Speaker/advisory role for Roche, AstraZeneca, MSD, BMS, Novartis, GSK, Astellas, Abbvie, Menarini, Pfizer, Sophia, Agilent, Merck, Amgen, Janssen, Lilly, BioGene Funding: Roche, AstraZeneca, Menarini, Pfizer, Agilent. J.A. receives advisory/speaker fees from Roche, Pfizer, MSD, Gilead, Menarini, Bayer, Lilly, Boehringer Ingelheim, Novartis, AstraZeneca Daiichi-Sankyo; travel Gilead, AstraZeneca Daiichi-Sankyo. J.A. and T.C-T have patents on using LCOR for therapeutic purposes (not related to this study). B.B. receives fees for consulting or advisory role with Lilly, Pfizer, MSD, AstraZeneca, Menarini, Gilead. Speakers’ bureau with Roche, MSD, Daichii Sankio, AstraZeneca, Novartis, Lilly, Gilead. Travel accommodation by Pfizer, Roche and Daichii Sankio.

## STAR★Methods

### Key resources table

**Table:** 

REAGENT or RESOURCE	SOURCE	IDENTIFIER
**Antibodies**		

IHC: Human TIM-3 Affinity Purified	R&D systems S.L	Cat#: AF2365; RRID: AB_355235
IHC: Polyclonal Rabbit anti-Goat, HRP	Dako	Cat#: P0449; RRID: AB_2617143
IF: TIM3 Monoclonal antibody	Proteintech	Cat#: 60355-1-Ig; RRID: AB_2881464
IF: Purified Mouse Anti-N-Cadherin	BD Biosciences	Cat#: 610920; RRID: AB_2077527
IF: Purified Mouse Anti-E-Cadherin	BD Biosciences	Cat#: 610182; RRID: AB_397581
IF: Recombinant Alexa Fluor® 488 Anti-Vimentin antibody	Abcam	Cat#: AB185030
IF: Purified Rat Anti-Mouse CD45R/B220	BD Biosciences	Cat#: 553084; RRID: AB_394614
IF: PE Armenian Hamster anti-mouse TCR γ/δ Antibody	BioLegend	Cat#: 118108; RRID: AB_313832
IF: Mouse β-catenin antibody (E−5)	Santa Cruz Biotechnology	Cat#: sc-7963; RRID: AB_626807
IF: Rabbit non-phospho (Active) β-catenin (Ser33/37/Thr41) (Clone D13A1)	Cell signaling	Cat#: 8814; RRID:AB_11127203
IF: mouse anti-GFP tag	Proteintech	Cat#: 66002-1-Ig; RRID: AB_11182611
IF: rabbit anti-mCherry	Abcam	Cat#: AB167453; RRID: AB_2571870
IF: Goat anti-Rabbit IgG (H + L) Cross-Adsorbed Secondary Antibody, Alexa Fluor™ 555	Invitrogen	Cat#: A-21428; RRID: AB_141784
IF: Goat anti-Rabbit IgG (H + L) Cross-Adsorbed Secondary Antibody, Alexa Fluor™ 647	Invitrogen	Cat#: A-21245; RRID: AB_2535813
IF: Goat anti-Mouse IgG (H + L), Superclonal™ Recombinant Secondary Antibody, Alexa Fluor™ 555	Invitrogen	Cat#: A28180; RRID: AB_2536164
IF: Goat anti-Mouse IgG (H + L), Superclonal™ Recombinant Secondary Antibody, Alexa Fluor™ 647	Invitrogen	Cat#: A32728; RRID: AB_2633277
IF: Goat anti-Rat IgG (H + L) Cross-Adsorbed Secondary Antibody, Alexa Fluor™ 555	Invitrogen	Cat#: A-21434; RRID: AB_2535855
IF: Goat anti-Armenian Hamster IgG (H + L) Highly Cross-Adsorbed Secondary Antibody, Alexa Fluor™ 647	Invitrogen	Cat#: A78967; RRID: AB_2925790
FC: Pe/Cy7 anti-TIM3 (Clone RMT3-23)	Biolegend	Cat#: 119716; RRID: AB_2571933
FC: APC/Cy7 anti-CD45 (Clone 30-F11)	Biolegend	Cat#: 103115; RRID: AB_312980
FC: FITCI anti-CD45 (Clone 30-F11)	Biolegend	Cat#: 103108; RRID: AB_312973
FC: Pe/Cy5 anti-CD3 (Clone 145-2C11)	Biolegend	Cat#: 100310; RRID: AB_312675
FC: PE/Dazzle594 anti-CD3e (Clone 145-2C11)	Biolegend	Cat#: 100347; RRID: AB_2564028
FC: PE anti-CD8 (Clone 53–6.7)	Biolegend	Cat#: 100708; RRID: AB_312747
FC: PercP/Cy5.5 anti-CD4 (Clone GK15)	Biolegend	Cat#: 100434; RRID: AB_893324
FC: FITCI anti-NK1.1 (Clone PK136)	Biolenged	Cat#: 108706; RRID: AB_313393
FC: Pe/Cy7 anti-CD220 (Clone RA3-6B2)	Biolegend	Cat#: 103201; RRID: AB_312986
FC: Texas Red anti-CD19 (Clone 6D5)	Biolegend	Cat#: 115501; RRID: AB_313636
FC: Pe/Cy7 anti-γδ TCR (Clone GL3)	Biolegend	Cat#: 118123; RRID: AB_11203530
FC: PE anti-hTIM3 (Clone F38-2E2)	Biolegend	Cat#: 345006; RRID: AB_2116576
FC: BV421 anti-hCD24 (Clone ML5)	BD Biosicences	Cat#: 562789; RRID: AB_2737796
FC: Pe/Cy7 anti-hCD44 (Clone IM7)	Biolegend	Cat#: 103028; RRID: AB_830785
sFC: BUV395 anti-CD8 (Clone 53–6.7)	BD Biosicences	Cat#: 565968; RRID: AB_2739421
sFC: BV421 anti-IL-17a (Clone TC11-18H10.1)	Biolegend	Cat#: 506925; RRID: AB_10900442
sFC: cFluor v547 anti-CD45 (Clone 30-F11)	Cytek	Cat#: R7-20571
sFC: BV750 anti-CD4 (Clone GK1.5)	Biolegend	Cat#: 100467; RRID: AB_2734150
sFC: AF488 anti-Gzmb (Clone QA18A28)	Biolegend	Cat#: 396423; RRID: AB_2924601
sFC: RB744 anti-CD3 (Clone 17A2)	BD Biosicences	Cat#: 570649; RRID: AB_3685926
sFC: PE-CF594 anti-CD69 (Clone H1.2F3)	BD Biosicences	Cat#: 562455; RRID: AB_11154217
sFC: Pe-Cy5 anti-Foxp3 (Clone FJK-16s)	Thermo	Cat#: 15-5773-80; RRID: AB_468805
sFC: APC anti-PD1 (Clone 29F.IAI2)	Biolegend	Cat#: 135210; RRID: AB_2159183
sFC: BUV395 anti-CD11b (Clone M1/70)	BD Biosicences	Cat#: 565976; RRID: AB_2721166
sFC: BUV737 anti-CD11c (Clone N418)	Invitrogen	Cat#: 367-0114-80; RRID: AB_2895934
sFC: BV785 anti-F4/80 (Clone BM8)	Biolegend	Cat#: 123141; RRID: AB_2563667
sFC: cFluor B584 anti-Ly6G (Clone 1A-8)	Cytek	Cat#: R7-20543
sFC: APC-Fire810 anti-Ly6C (Clone HK1-4)	Biolegend	Cat#: 128055; RRID: AB_2910291
WB: Mouse monoclonal anti-vinculin (clone 7F9)	Santa Cruz	Cat#: sc-73614; RRID: AB_1131294
WB: anti-phospho GSK3 alpha/beta (S21/9)	Cell Signaling	Cat#: 9331S; RRID: AB_329830
WB: Goat anti-Rabbit IgG H&L (HRP)	Abcam	Cat#: AB6721; RRID: AB_955447
WB: Rabbit anti-Mouse IgG H&L (HRP)	Abcam	Cat#: AB6728; RRID: AB_955440
*In vivo*: anti-IgG2b isotype (Clone MPC-11)	BioXCell	Cat#: BE0086; RRID: AB_1107791
*In vivo*: anti-IgG1 isotype (Clone TNP6A7)	BioXCell	Cat#: BP0290; RRID: AB_2687813
*In vivo*: anti-mouse CD4 (Clone GK1.5)	BioXCell	Cat#: BE0003-1; RRID: AB_1107636
*In vivo*: anti-mouse CD8 (Clone YTS169.4)	BioXCell	Cat#: BE0117; RRID: AB_10950145
*In vivo*: anti-mouse TCRγδ (Clone UC7-13D5)	BioXCell	Cat#: BE0070; RRID: AB_1107751
*In vivo*: anti-rat Kappa immunoglobulin (Clone MAR 18.5)	BioXCell	Cat#: BE0122; RRID: AB_10951292
*In vivo*: anti-mouse Ly6G (Clone 1A8)	BioXCell	Cat#: BP0075-1; RRID: AB_1107721
*In vivo*: anti-mouse IL-1b	BioXCell	Cat#: BE0246; RRID: AB_2687727
*In vivo*: anti-mouse TIM3, InVivoPlus (Clone RMT3-23)	BioXCell	Cat#: BP0115; RRID: AB_10949464
*In vivo*: Ultra-LEAF anti-mouse CD20 (Clone SA2711G2)	BioLegend	Cat#: 152116; RRID: AB_2629619
*In vivo*: anti-asialo GM1	Wako Chemicals	Cat#: 98610001; RRID: AB_516844

**Bacterial and virus strains**		

*Tim3*-mCherry-IRES-Nluc	VectorBuilder	VB211227-1106hky
Cre reporter (EF1a-LoxP-DsRed-STOP-LoxP-eGFP)	Addgene	Cat#: 62732
*Tim3*-CreERT2-Neo	VectorBuilder	VB230412-1204pkn
Px330-mCherry	Addgene	Cat#: 98750
pSpCas9(BB)-2A-GFP (PX458)	Addgene	Cat#: 48138

**Chemical, peptides and recombinant proteins**		

BSA Bovine Serum Albumin	Sigma-Aldrich Quimica S.L.	Cat#: A790_6
Collagenase	Sigma-Aldrich Quimica S.L.	Cat#: C2674
DNase I	Merck Life Sciences S.L.U	Cat#: D5025
Hyaluronidase type 4	Sigma-Aldrich Quimica S.L.	Cat#: H3506
Percoll	ACEFE S.A.U.	Cat#: 17-0891-02
Recombinant mouse IL-2	TEBU-BIO SPAIN S.L.	Cat#: 212-12B
Recombinant mouse IL-15	TEBU-BIO SPAIN S.L.	Cat#: 210-15

**Critical commercial assays**		

TCRγ/δ + T cell Isolation kit	Milteny Biotec	130-092-125
Gentlemac C Tubes	Milteny Biotec	130-093-237
LD selection column	Milteny Biotec	130-042-901
Dynabeads Mouse T-activator CD3/CD28	Gibco	Cat·: 11456D

**Experimental models: Organisms/Strains**		

Balb/cAnNCrl, H2^d^	Charles Rivers	Strain code: 028
NOD.Cg-Prkdc^SCID^ Il2rg^tm1Wjl^/SzJ	Charles Rivers	Strain code: 614
C57BL/6J	Animal Facility	Strain code: 632

**Experimental models: Cell lines**		

EpRas	Y.Kang, Princeton	
4T07	Y.Kang, Princeton	
4T1	Y.Kang, Princeton	
AT3	Y.Kang, Princeton	
HEK	ATCC	

**Experimental models: Human Samples**		

Primary Breast Cancer tumors	MAR Biobanc, Barcelona Fundación Jiménez Díaz Biobank, Madrid Clinic Hospital Biobank, Valencia	
Primary and metastatic samples	Geicam	

**Oligonucleotides**		

m*Gapdh* qRT-PCR primer FW	Integrated DNA Technologies	AGGTCGGTGTGAACGGATTTG
m*Gapdh* qRT-PCR primer REV	Integrated DNA Technologies	TGTAGACCATGTAGTTGAGGTCA
m*Hmbs* qRT-PCR primer FW	Integrated DNA Technologies	CGGGAAAACCCTTGTGATGC
m*Hmbs* qRT-PCR primer REV	Integrated DNA Technologies	CTCAGAGAGCTGGTTCCCAC
m*Tim3* qRT-PCR primer FW	Integrated DNA Technologies	AGACATCAAAGCAGCCAAGGT
m*Tim3* qRT-PCR primer REV	Integrated DNA Technologies	TCCGTGGTTAGGGTTCTTGG
m*Pou5f1* qRT-PCR primer FW	Integrated DNA Technologies	CCCGGAAGAGAAAGCGAACT
m*Pou5f1* qRT-PCR primer REV	Integrated DNA Technologies	CCAAGCTGATTGGCGATGTG
m*Nanog* qRT-PCR primer FW	Integrated DNA Technologies	GATTCAGGGCTCAGCACCA
m*Nanog* qRT-PCR primer REV	Integrated DNA Technologies	AAGGCTTCCAGATGCGTTCA
m*Sox2* qRT-PCR primer FW	Integrated DNA Technologies	AGAGCTAGACTCCGGGCGATG
m*Sox2* qRT-PCR primer REV	Integrated DNA Technologies	ACCCAGCAAGAACCCTTTCCTCG
m*Snai2* qRT-PCR primer FW	Integrated DNA Technologies	CTCACCTCGGGAGCATACAG
m*Snai2* qRT-PCR primer REV	Integrated DNA Technologies	GACTTACACGCCCCAAGGATG
m*IL1b* qRT-PCR primer FW	Integrated DNA Technologies	GCAACTGTTCCTGAACTCAACT
m*IL1b* qRT-PCR primer REV	Integrated DNA Technologies	ATCTTTTGGGGTCCGTCAACT
IL1b gRNA 1	Integrated DNA Technologies	ACAAGGAAGCTTGGCTGGAG
IL1b gRNA 2	Integrated DNA Technologies	GGCATTTCACAGTTGAGTTC
IL1b gRNA 3	Integrated DNA Technologies	GTCCGTCAACTTCAAAGAAC

**Deposited data**		

Raw transcriptomic data: EpRas metastasis from different mouse strains	This Paper	GEO: GSE260481
Raw transcriptomic data: 4T07-Ctrl and *Tim3*-KD from lung and liver metastasis	This Paper	GEO: GSE260480
scRNA-seq of CD45^+^ cells from 4T07 liver metastasis	This Paper	GEO: GSE260482

**Software and algorithms**		

FlowJo 10		
Galore		
Plolty R package (v4.9.1)		
Prism 8		
R Studio		
**Other**		

FACSAria Cell Sorter	BD Bioscience	UPF-PRBB Facility
S8 Cell Sorter	BD Bioscience	
Fortessa	BD Bioscience	
Aurora	BD Bioscience	
Gentle MACS Octo Dissociator	Milteny Biotec	Cat#: 130-096-427
QuantStudio 12K	Applied Biosystems	UPF-PRBB Facility
Pump perfusion	BIOGEN CIENTIFICA S.L.	Cat#: P-DKIT
Nikon Eclipse		EMBL-PRBB Facility
TCS SP5 Confocal Microscope	Leica	

### Experimental model and subject details

#### Mice

Mice were housed in pathogen-free conditions at the animal facility of the Barcelona Biomedical Park Research (PRBB). All animal procedures performed in this study were approved by the Ethical Committee for Animal Research of the PRBB and by the Catalonia Government. Euthanasia was applied when animal health was compromised.

#### Cell lines

Cancer cell lines (4T07, 4T1, 66cl4, EpRas, MDAMD231 and AT3) were obtained from Y.Kang at Princeton University and cultured in DMEM media supplemented with 10% of Fetal Bovine Serum (FBS), 2mM L-Glutamine (Glu) and 1% Penicillin/Streptomycin (P/S). HEK293T cells were obtained from ATCC. Full splenocytes and CD8 T cells extracted from OT-I mice were cultured in RPMI 1640 media (Life Technologies, Cat.21875-034) supplemented with 10% of Fetal Bovine Serum (FBS) and 1% Penicillin/Streptomycin (P/S).

#### Human samples

Surgical resection specimens from primary breast tumors obtained from Hospital del Mar Biobank (MARBiobanc, Barcelona, Spain), Fundación Jiménez Díaz Biobank (Madrid, Spain) and Valencia Clinic Hospital Biobank (Valencia, Spain) have the approval from the Ethical Committee of Clinical Investigation. All individuals gave their informed consent before inclusion.

### Method details

#### Animal studies

In this study, Balb/c, C57BL/6J, and NOD.Cg-Prkdcscid Il2rgtm1Wjl/SzJ (NSG) mouse strains were used. For metastasis assays, mice were anaesthetized using medetomidine (1 mg/Kg) and ketamine (100 mg/Kg) intraperitoneal administration. Mice were shaved and intracardiac injections were performed with 20,000 cells resuspended in 100 μL of sterile PBS 1X injected in the left ventricle of the mice using a 26G insulin syringe. After intracardiac injection, 100 μL of luciferin (DISMED S.A, LUCK-1) was administered via retroorbital injection to ensure systemic bloodstream delivery, while mice were under isoflurane inhalation (3.5% isoflurane + O2, 0.8L/min). Metastatic growth was measured by bioluminescence (BLI) acquisition using the IVIS system, once or twice per week depending on the experiment for 4–5 weeks. All images were acquired using 1 min of exposure and binning 4. Photon flux quantification was performed with the same ROI for all timepoints using Living-Imaging Software (Perkin Elmer 4.7.3). Orthotopic mammary fat pad (MFP) injection was performed into the fourth mammary gland in the right and left side. After isoflurane inhalation, the incision was done to expose the transplantation site. Cells were resuspended in 1:1 PBS:Matrigel and 10 μl were injected just above the lymph node using a 26 gauge-Hamilton syringe. Wound clips were used to close the incision site. For tumor initiating capacity (TIC) examination, immunocompromised NSG female mice were orthotopically transplanted with series of limiting- cell dilution assays (LDA). Tumor growth rate was measured weekly using a calibrated digital caliper (Merck, Z503576-1EA). For spontaneous metastasis experiments, tumors were surgically resected at 7 × 7mm and metastasis appearance was controlled by BLI measurements. Only upper body images were shown to avoid masking of BLI from primary tumor regrowth. Liver metastasis assay was performed using intraportal vein injection. After anesthesia administration, the surgical area was cleaned with an alcohol pad. Mice were placed in a supine position and the incision was performed in the ventral left side. Using sterile cotton swabs, large and small intestines were pulled out into a gauze pad. Once portal vein was visualized, 10 μL of cells resuspended in PBS 1X were injected using a customized Hamilton of 32G. The hemostatic gauze was held in the injection site until blood flow ceased completely. Then, internal organs were placed back into the abdominal cavity and the peritoneal area was closed with 4-0 vicryl suture. The skin was closed using sterile clips. Liver metastasis growth was measured using bioluminescence acquisition in the IVIS system. After all procedures, buprenorphine (0.05 mg/kg) was injected for 3 days to control post-procedural pain. For organ analysis, animals were euthanized after luciferin administration and ex-vivo organs were placed into a petri dish for BLI measurement using IVIS system. After image acquisition, organs were digested for tumor cells or immune cells isolation according to the follow-up procedures.

#### *In vivo* therapies and specific cell depletion/neutralization

For *in vivo* depletion, 250 μg of antibody were administered by intraperitoneal injection every 4 days during the 3–4 weeks of experiments. Anti-mouse CD4 (Clone GK1.5, Cat. BE0003-1; BioXCell), anti-mouse CD8 (Clone YTS169.4, Cat. BE0117, BioXcell); Ultra-LEAF Purified Rat Ig2b Isotype Control (Clone TRK4530, Cat. 400671, BioLegend); Ultra-LEAF Purified anti-mouse CD20 (Clone SA271G2, Cat. 152116, BioLegend); anti-rat IgG2a isotype control (Clone 2A3, Cat. BP0089, BioXCell); anti-TCRγδ (Clone UC7-13D5, Cat. BE0070, BioXCell); anti-Rat Kappa immunoglobulin (Clone MAR 18.5, Cat BP0290, BioXCell); anti-mouse Ly6G (Clone 1A8, cat BP0075-1, BioXCell) were used. For NK depletion, 100 μL of anti-asialo-GM1 (Wako Chemicals Cat. 98610001) were administered. Immune cell depletion was confirmed by labelling peripheral blood and it was analyzed by flow cytometry. For TIM3 blockade therapy, anti-mouse TIM3 (InVivoPlus Clone RMT3-23, Cat. BP0115, BioXcell) was used for intraperitoneal injection.

#### Cell lines treatments

For this study, FH353 (β-catenin inhibitor, Merck Millipore CAS108409-83-2); mIL-2 (Tebu-Bio Spain S.L., Cat.212-12B) were used. For the lineage tracing; *in vitro*, cell lines were induced with 1 μM of 4-OHT (Merck, H7904). *In vivo*, mice were injected via i.p. with 1.2 mg of tamoxifen (Merck, T5648).

#### Molecular cloning and plasmids

The FiG (Firefly-IREs-GFP) plasmid was kindly provided by Y.Kang Lab. For mouse *Tim3* knock-down (KD) experiments, shRNAs were purchased from Sigma-Aldrich (nos. TRCN0000099986, TRCN0000099987, TRCN0000099988, TRCN0000099989) in pLKO-Puro lentiviral backbone along with pLKO.1 control vector targeting a scramble RNA. For overexpression (OE) experiments, mouse *Tim3* was amplified by PCR and inserted into pLEX-MCS plasmid after SpeI and AgeI digestion (NEB). For the lineage tracing, the Cre Reporter plasmid (EF1a-LoxP-DsRed-STOP-LoxP-eGFP) was purchased from Addgene (Plasmid #62732). The CreERT2 under the promoter of *Tim3* was generated by VectorBuilder. For 4T07 IL-1β KO generation, three independent guides (STAR table) were annealed and cloned into digested pSpCas9(BB)-2A-GFP (px458) and pSpCas9(BB)-2A-mCherry (px330) with FastDigest Bpil (Fisher Scientific, catalog no. FD1014). Two pairs of guides cloned into GFP and mCherry Cas9 plasmids respectively were co-transfected into 4T07 through electroporation (1 pulse of 1700 V and 20 ms width). After 48h, double positive cells were sorted in single-cell and collected into 96-well plates. Il1β KO clones were validated by PCR and Sanger sequencing.

#### Luciferase-based reporter assay

*Tim3*-reporter was designed using mCherry under the promoter of *Tim3* followed by internal ribosomal entry site (IRES) and nanoluciferase (Nluc). This construct was generated by VectorBuilder. For the *Tim3*-reporter assay, *in vitro* mCherry mean fluorescence intensity was measured by flow cytometry. *In vivo*, nano-light substrate (Promega) was retro-orbitally injected to the mice and BLI was measured by IVIS. For Firefly-luciferase metastasis tumor bulk assessment, after 6h of nanolight administration, luciferin (Dismed S.A.) was retro-orbitally injected to obtain total metastatic quantification.

#### Lineage tracing

CRE reporter construct (EF1a LoxP-DsRed-STOP-LoxP-eGFP) labels cells in red, and switch to green upon LoxP excision. *Tim3*-CreERT2 construct drives CreERT2 expression under the promoter of *Tim3*. CreERT2 recombinase activity depends on Tamoxifen binding and leads to the excision of DsRed from the EF1a-LoxP-DsRed-STOP-LoxP-eGFP cassette, thereby permanently labeling cells in green (eGFP). TIM3^-^ cancer cells have red fluorescence by expressing DsRed. *Tim3*^*+*^ cancer cells have green fluorescence by expressing eGFP, and not DsRed anymore. Short-term induction: The system is activated *in vitro* 48h before injection and 1.2 mg of TAM during the first 3 days of metastatic seeding. Treatment withdrawal at day 3 and organs were harvested at day 20. Long-term induction: The system is activated *in vitro* 48h before injection and 1.2 mg of TAM during the entire experiment duration, up to 20 days. Organs were harvested at the end point of the experiment.

#### Viral production and infection of cell lines

HEK293T cells were transfected with PEI with lentiviral (Lv) packaging plasmid (VSV-G) and gag-pol plasmid (pCMV-R8.91) following the second-generation Lv protocol. One day after transfection, the media was refreshed, and 24h and 48h later, supernatants were collected, centrifuged and purified through 0.45 μm filter. Viruses were stored at −80°C. For lentiviral infection, 100.000 cells were plated in 6-well plates with 1mL of Lvs conditional media. Antibiotic selection or GFP sorting was applied based on plasmid requirements after 48h post-infection.

#### Immunofluorescence (IF)

##### IF in cell lines

Cell lines were seeded in cover glasses O/N and then washed twice with PBS 1X supplemented with 0.25% Triton X-100. Fixation was performed with 4% PFA during 1h at RT. Blocking buffer (PBS 1X supplemented with 5% of NGS) was added for 1h with shaking. After washing, primary antibody was diluted in blocking buffer and incubated O/N at 4°C. The next day, after 3 washes of PBS, secondary antibody was added for 1h shaking at RT, protected from light. Finally, cover glasses were mounted with Fluoromount with DAPI. Images were acquired with a Nikon Eclipse Ni-E microscope and analyzed with ImageJ.

##### IF in tissues

For tissue fixation, 30mL of PBS 1X solution was perfused followed by 30mL of formalin. After tissue clearance, livers were resected and included in paraffin blocks to continue the appropriate staining protocol. After mice perfusion, harvested organs were fixed in 4% PFA O/N at 4°C. Once washed with PBS, organs were embedded into paraffin blocks and 5 μm sections were cut. Liver slides were placed at 50°C-60°C until paraffine starts to sweat followed by deparaffinization in xylene. Hydration was performed with ethanol gradients and the antigen retrieval was done in pre-heated Sodium Citrate Buffer 0.01M pH 6.0 in a pressure cooker for 20 min. For IHF, after incubation with 3% of sudan black, slides were dehydrated and blocking was performed in PBS supplemented with 10% FBS at RT for 1h. Then, primary antibody was incubated O/N at 4°C followed by secondary antibody incubation after washing. Finally, Fluoromount with DAPI was used to stain and visualize the image.

##### Lineage tracing analysis

Livers were directly embedded in paraffin and 5 μm sections were cut to proceed for fluorescence microscopy. Tissues were stained with DAPI, anti-GFP and anti-DsRed. Criteria for lineage positivity: metastatic lesions were considered positive when GFP^+^ cells were detected, induced by CreERT2, independently of the intensity of the green signal and residual dsRed signal. Negative lineage was considered with DsRed detection and no GFP detection. The percentage of positive and negative *Tim3* lineage was calculated based on the total number of metastatic lesions detected across all mouse livers harvested.

##### IF in human samples

Double immunofluorescence for co-expression analysis was performed on two consecutive 3-μm tissue sections. Heat antigen retrieval was carried out in pH9 EDTA-based buffered solution in a Dako Link platform. A goat polyclonal anti-TIM3 (AF2365), and a mouse monoclonal anti-Mammaglobin (clone 304-1A5, GA074) antibodies were used. Appropriate Alexa Fluor 568 and 488 -conjugated rabbit anti-goat IgG and anti-mouse IgG antibodies (Invitrogen, Thermo Fisher Scientific; diluted 1:700) were applied. Sections were counterstained with 4′,6-diamidino-2-phenylindole dihydrochloride (DAPI; Abbott Molecular) to visualize cell nuclei. All incubations were performed at room temperature in Autostainer platform (Dako Agilent). Staining was evaluated by two investigators (F. Rojo and S. Pérez-Buira) using a Cri Nuance FX Multispectral Imaging System (PerkinElmer).

#### Immunohistochemistry (IHC)

Immunostaining was carried out using 3-μm sections. Heat antigen retrieval was carried out in pH9 EDTA-based buffered solution in a Dako Link platform. Endogenous peroxidase was quenched. A goat polyclonal anti-TIM3 antibody (AF2365) was used for 20 min at room temperature, with high antibody concentration (1:80 dilution), followed by incubation with a polymer coupled with peroxidase (Flex+; Dako). Sections were then visualized with 3,3′-diaminobenzidine (DAB) and counterstained with haematoxylin. TIM3 expression in breast cancer specimens was evaluated by two senior pathologists (FR and LC), based on published studies.^93^ This work was carried out in accordance with Reporting Recommendations for Tumor Marker Prognostic Studies (REMARK) guideline. Histological tumor compartments and characteristics were cataloged by microscopic morphological evaluation in hematoxylin eosin sections obtained sequentially to IHC staining. The categorization was done by two experienced pathologist that considered different cell properties such as nuclear size, margin, cellular pleomorphism, nucleoli forms, and cell decohesion. The malignant epithelial cells were distinguished using mammaglobin staining by IF, but also from H&E sections based on moderate to large increase in size and variability of nuclei, chromatin in vesicles with prominent nucleoli and abundant eosinophilic cytoplasm. Other features including foamy or granular morphology, deformed architecture of sheets of cells or individual tumor cells in absence of myoepithelial basal cell lining. The immune cells observed consisted of lymphocytes, plasma cells, and macrophages, each with distinct morphological features. Tumor-associated Macrophages (TAMs) appeared as larger cells with a round or oval shape, featuring an eccentrically positioned oval or indented nucleus and cytoplasm with a foamy texture. Stromal and intratumoral Lymphocytes (TILs) exhibited a round to ovoid shape, characterized by a small, uniform spherical nucleus with condensed chromatin, no visible nucleoli, and a minimal pale cytoplasm. Cancer-associated fibroblasts (CAFs) were characterized by elongated, rounded nuclei with faint, uniform chromatin and no visible nucleoli. Their cytoplasm was poorly defined, eosinophilic, and spindle-shaped. These fibroblasts were observed as isolated cells within stromal regions containing collagen.

#### Proximity ligation assay (PLA)

Cells were plated at the bottom of the cover glasses and then fixation and permeabilization was performed according to the immunofluorescence protocol mentioned above. For proximity ligation assay (PLA), Naveni TriFlex Cell MR kit was used (TF.MR.100, Navinci) following manufacturer instructions. In the primary antibody incubation, mouse anti-Tim3 (Proteintech) and rabbit anti-p85 (4257T, Cell signaling) were used. The proximity signal was detected using Cy5 filter.

#### Phospho-kinase array

For the phosphor-kinase array, the Proteome profiler human phospho-kinase array kit (ATY003C, R&D Systems) was used following the manufacturer protocol. Briefly, cultured cells were collected in lysis buffer and incubated on the nitrocellulose membranes to bind the specific target proteins from the sample. Then, serial steps of washings and antibody cocktails were applied to detect up to 37 human kinase phosphorylation. Finally, chemiluminescent detection reagents were used to visualize the reaction. The intensity of the membrane was quantified using ImageJ.

#### Western blot

For protein analysis, cells were lysed using RIPA buffer (1% SDS, 20 mM Tris-HCl (pH 7.5), 150 mM NaCl, 1 mM Na_2_EDTA, 1 mM EGTA 1%, NP-40, 1% sodium deoxycholate) supplemented with phospho-STOP (Sigma) and protease inhibitor cocktail cOmplete (Merck). Extracted protein was quantified using Bradford assay and 30 μg were run in SDS-PAGE gels. After electrophoresis, samples were transferred to PVDF membranes (Millipore) and blocked in 5% BSA in TBS-T. Primary incubation was performed O/N at 4°C and secondary incubation was done for 1h at R.T. Antibodies used are listed in the key resources table. Protein detection was performed using Alliance Q9 (UVITEC) chemiluminescence imager.

#### Metastatic tissue digestions for tumor cell follow-up procedures

Tissue digestion to obtain tumor cells: after mice euthanasia, metastatic organs were collected into ice-cold DMEM. Lung and liver were mechanically digested with scalpels. The brain was smashed into a cell strainer using a 15 mL dounce and then an isotonic Percoll solution was added for the isolation of tumor cells. Then, digested organs were incubated with DMEM supplemented with collagenase A and hyaluronidase for 2h shaking at 37°C. After enzymatic digestion, organs were treated with dispase/DNaseI solution. Cells were also incubated with trypsin and red blood lysis buffer to finally obtain single cells purified through 40 μm cell strainers. Finally, cells obtained were sorted for GFP positivity. Immediately after, RNA was isolated for gene expression analysis and RNA-sequencing procedures.

#### Gene-expression analysis

RNA was isolated from cells using the RNAeasy Mini Kit (QIAGEN) and it was reverse transcribed into cDNA using the High-Capacity cDNA Reverse Transcription Kit (Life Technologies). Light Cycler 480 SYBR Green I Master (Merck) was used to perform RT-qPCR and QuantStudio 12K Flex software to collect the amplification data. *GAPDH* and *HMBS* levels were used to normalized gene expression data. Primers used for gene mRNA expression detection are listed in the key resources table.

#### Flow cytometry and cell sorting

After trypsinization, cells were counted and 100.000 cells were centrifuged at 300G for 5 min for tumor cells and 600G during 7 min for immune cells. The pellet was resuspended in 100 μl of FACS buffer (PBS 1X, 10% FBS) and the corresponding antibody was diluted according to the specific titration. The reaction was incubated for 30 min at 4°C in rotation. After washing, tumor cells were analyzed in the Fortessa flow cytometer (BD Biosciences) and by FlowJo software v10.4.2 (FlowJo). For cell sorting, the standard flow cytometry protocol was used in order to isolate the desired population and cells were collected in 15 mL tubes using FacsAria (BD).

##### Spectral flow cytometry

Digested micrometastasis brought to single-cell suspension were measured by the Cytek Aurora Spectral analyzer 5L (Cytek Biosciences). The gating strategy for each subpopulation is specified in Figure S7, where 11 antibodies were used for lymphoid cell detection and 8 antibodies for myeloid cell detection, according to the specific titration. The panel of antibodies details are indicated in the STAR Methods table (sFC). For the detection of intracellular staining of IL-17, Gzmb and Foxp3, single cell suspensions were stimulated with the stimulation cocktail containing Phorbol- 12-myristat-13-acetate plus Ionomycin and Brefeldin A (from Biolegend) for 4h at 37°C. Cells were then first incubated with FcγIII/II receptor (CD16/CD32) and True-Stain Monocyte blocking antibodies for 10 min at 4°C followed by surface staining with antibodies for 20 minutes at 4°C. Fixable viability dye live/dead blue (from Invitrogen) was used to exclude dead cells. For intranuclear staining, cells were fixed and permeabilized with Foxp3/Transcription Factor kit (from Invitrogen) according to manufacturer’s protocol, followed by 1h of incubation at 4°C with intracellular antibodies. All data were collected on an Aurora 5L (Cytek) instrument and analyzed with FlowJo 10.7.1 software (TreeStar).

#### Tumorsphere assay

Cells were grown in mammosphere media as previously described^94^ with 1:1 DMEM (D5796, Merck) and F-12 w/L-Glutamine (BE12-615F, Cultek) supplemented with 50× B-27, 20 ng/mL EGF (AF-100-15-B, Tebu-Bio) and 20 ng/mL bFGF (100-18B-B, Tebu-Bio) in 24-well ultra-low attachment plates (Ref.3473, Cultek) at a density of 5.000 cells per well. Sphere formation was quantified at day 5 after seeding.

#### γδ T cell *in vitro* isolation, expansion and activation

Spleens from Balb/c mice were harvested and processed mechanically to obtain a single-cell suspension. T cells were separated using the mouse TCRγ/δ+ T Cell Isolation Kit (Milteny Biotec, 130-092-125) and LD columns (Milteny Biotec, 130-042-901). γδ T cells were further isolated by spectral cell sorting (S8 Cell Sorter, BD) using the following markers: CD45 FITC^+^, γδ TCR PE/Cy^+^, CD3 PE/Dazzle 594^+^. For expansion and activation, sorted γδ T cells were cultured at 1 million cells/mL in a 96-well plate with RPMI 1640 media supplemented with 20 ng/mL mouse IL-2 (TEBU-BIO SPAIN S.L., 212-12B), 10 ng/mL mouse IL-15 (TEBU-BIO SPAIN S.L., 210-15), Dynabeads Mouse T-activator CD3/CD28 (Gibco, 11456D) at a ratio of 1 cell: 1 bead, 10% FBS, 1% Penicillin/Streptomycin and 50 μM 2-β-mercaptoethanol. At day 4, Dynabeads Mouse T-activator CD3/CD28 were removed from the media, and cells were kept in culture for three more days at 0,7 million cells/mL^64^^,^^67^

#### Tumor and γδ T cell coculture assays

For co-culture assays of 4T07 tumor cells and γδ T cells, one day before co-culture, 4T07 cells were plated at 25,000 cells/well in 12-well plates with RPMI 1640 media supplemented with 10% FBS, 1% Penicillin/Streptomycin and 50 μM 2-β-mercaptoethanol. Then, 5 μg/mL anti-mouse IL-1β blocking antibody or control mouse IgG (sc-2025, Santa Cruz Biotechnology) were added to the media when indicated. After the O/N, the media was removed and γδ T cells were added at an effector-to-target ratio of 10:1 in media without antibodies. Cells were co-cultured for 24h at 37°C. Then, the media containing the γδ T cells was collected, and γδ T cells were stimulated with the Cell Activation Cocktail (BioLegend, 423303) for 4h at 37°C for flow cytometry analysis. Then, cells were stained for the extracellular markers CD3 and γδ TCR and the intracellular marker IL-17A. Viability was determined using the LIVE/DEAD Fixable Blue Dead Cell Stain Kit (Invitrogen, L23105) and spectral flow cytometry was used.

#### Clinical samples and data analysis

Formalin-fixed paraffin-embedded (FFPE) blocks from primary breast cancer and matched biopsy specimens of recurrent or metastatic tumors were obtained from all participants in the ConvertHER study in 31 clinical sites (De Dueñas et al. BCRT 2014). Samples were stained for TIM3 (IHC). TIM3 status was evaluated in tumor cells and in the tumor microenvironment. Samples were considered TIM3^+^ when at least 1% or more cells (specific of the different cellular compartments analyzed) were positive. TIM3 scoring percentage includes the % quantity of TIM3 positive cells.

Surgical resection specimens from primary breast tumors obtained from Hospital del Mar Biobank (MARBiobanc, Barcelona, Spain), Fundación Jiménez Díaz Biobank (Madrid, Spain) and Valencia Clinic Hospital Biobank (Valencia, Spain). Tumor specimens from FFPE blocks were retrospectively selected from consecutive breast cancer patients diagnosed between 1998 and 2000, which had the following criteria: infiltrating carcinomas, operable, no neoadjuvant therapy, enough available tissue and clinical follow-up. TNM (tumor–node–metastasis) staging was classified using the American Joint Committee on Cancer (AJCC) staging system. Histological grade was defined according to Scarff–Bloom–Richardson modified by Elston. ER and PR were determined by IHC (SP1 and PgR636 clones, respectively) establishing positivity criteria in ≥1% of nuclear tumor staining. HER2 amplification was assayed by FISH (Pathvysion). Ki-67 was studied by IHC (MIB1 clone; Dako). TIM3 was evaluated by IHC. The study was approved by the Ethics Committee of the Hospital del Mar. 260 infiltrating carcinomas were collected. Tissue microarrays (TMA) were constructed as reported. Annotated data of breast cancer patients were analyzed with SPSS software (v29.0.2.0) for survival and regression tests. Patient stratification was performed by TIM3 expression. For statistical significance, Log Rank (Mantel-Cox) test was applied. Overall survival (OS) was defined as the time from the date of surgery to the date of death from any cause or last follow-up. Disease-free survival (DFS) was considered from the date of surgery to the date of any primary, regional or distant recurrence, as well as the appearance of a secondary tumor or DCIS. Univariate analysis was based on the Kaplan–Meier OS and DFS curves using the log-rank test; all predictors with *p*-values <0.1 were used in multivariate analysis using the Cox proportional hazards model. All the statistical tests were conducted at the two-sided 0.05 level.

#### RNA sequencing and bioinformatic analysis

Total RNA extracted from tumor cells of metastatic organs was quality-checked with BioAnalyzer prior to sequencing. Poly-A sequencing was selected for library preparation, and samples were sequenced using the Illumina Hi-Seq 2500 platform with 1 × 50-bp settings at the Centre of Genomic Regulation (CRG) using the paired-end method. Quality checking of raw data (fastq files) was performed with FastQC software (v.0.11.9). Estimation of ribosomal RNA in the raw data was obtained with riboPicker74. Raw reads were trimmed using TrimGalore (v.0.6.6) with a quality cutoff of 30. The trimmed data were then aligned with the STAR (v.2.7.8a) mapper75 to the Mus musculus genome (Gencode release M24 of the GRMm38/mm10 assembly: https://www.gencodegenes.org/mouse/release_M24.html). The raw count of reads per gene and per sample were obtained with STAR (quantMode TranscriptomeSAM GeneCounts option). The R/Bioconductor package DESeq2 (v.1.30.1) was used to assess differential expression between experimental groups (Wald statistical test plus false discovery rate (FDR) correction). Genes for which the sum of raw counts across all samples was <1 were discarded. Genes are considered differentially expressed if their absolute log2 fold change was >1 and their adjusted *p*-value <0.05.

#### Gene set variation analysis (GSVA)

Single gene set variation analysis (GSVA package v.1.50.0) was performed using TCGA-BRCA-TNBC clinical data obtained from cBioportal formed by 237 patients. Stemness (GUPTA-UP) signatures and β-catenin related signatures were interrogated together with *TIM3* levels in the above-mentioned clinical dataset. The Rho correlation and *p*-value were calculated to assess test significance.

#### Gene set enrichment analysis (GSEA) and gene ontology (GO)

For gene set enrichment analysis (GSEA), two different datasets were used. First, immunoediting RNA-sequencing data grouped by different hosts as conditions (ID and IC) for 3 different organs using EpRas cell line. Second, *Tim3*^+^-Ctrl and *Tim3*-KD 4T07 cells from lung and liver samples. Both datasets were interrogated for HALLMARKS genesets (MSigDB) and stemness signatures: mammary stem cells (LIM_Mammary_Stem_Cell_UP) formed by 480 genes,^27^ malignant stem cells (Wong CSC),^43^ EMT (Hallmarks EMT UP) formed by 200 genes,^95^ and liver cancer stem cells formed by 47 genes (Yamashita Liver CSCs).^44^ All datasets were interrogated with 1000 random permutations of their phenotype labels to obtain a normalized enrichment score (NES) and *p* value. Transcriptomic data was interrogated for gene ontology (GO) analysis using the upregulated and significant genes obtained in the RNA-seq experiments using the Enrichr software.

#### Single cell RNA sequencing

For immune cell isolation, a piece 5 × 5 mm of the liver was chopped with scalpels and then incubated with digestion buffer composed by Hanks balanced solution (Gibco-HBSS, Ref.14025134), Collagenase III 0.3 mg/mL (C2674, Sigma-Aldrich Quimica S.L) and DNaseI 10 U/mL (D5025-150, Sigma-Aldrich Quimica S.L.). The samples were incubated 15 min at 37°C using the Gentle MACS dissociation machine in gentlemacs C tubes (130-093-237, Miltenyi Biotec S.L.). Cells were strained trough 100 μm and 40 μm cell strainers and centrifuge at 50G for 1 min. Immune cells in the supernatant were stained for CD45 marker and sorted using FacsAria (BD). Each sample for sequencing was a pool of two independent mice.

Cells were centrifuged at 700 rcf during 5 min at 4° in order to bring the cell concentration to 300–-1000 cells/μL. Cell concentration and viability were determined using a TC20™ Automated Cell Counter (Bio-Rad Laboratories, S.A) upon staining the cells with Trypan blue. Cells were partitioned into Gel Bead-In-Emulsions (GEMs) by using the Chromium Controller system (10× Genomics), with a target recovery of 5000 total cells per sample. cDNA sequencing libraries were prepared using the Next GEM Single Cell 3′ Reagent Kits v3.1 (10× Genomics, PN-1000268), following manufacturer’s instructions. Briefly, after GEM-RT clean up, cDNA was amplified during 12 cycles and cDNA quality control and quantification were performed on an Agilent Bioanalyzer High Sensitivity chip (Agilent Technologies). cDNA libraries were indexed by PCR using the PN-1000215 Dual Index Kit TT Set A. Size distribution and concentration of 3′ cDNA libraries were verified on an Agilent Bioanalyzer High Sensitivity chip (Agilent Technologies). Finally, sequencing of cDNA libraries was carried out on an Illumina NovaSeq 6000 using the following sequencing conditions: 28 bp (Read 1) + 10 bp (i7 index) + 10 bp (i5 index) + 90 bp (Read 2), to obtain approximately 20–30.000 reads per cell.

For raw data processing, Reads 2 with a white-listed cell barcode extracted from their corresponding Reads 1 were selected, trimmed using TrimGalore (v.0.4.3) with default parameters and mapped using STAR (v.2.7.7a) with default parameters to the mouse genome (GRCm38, Ensembl 102). Only reads mapping to gene bodies (exons or introns) were used for downstream analysis. Reads mapping simultaneously to an exon and to an intron were assigned to the exon, as described in.^96^ Only cells with more than 1000 μmi-corrected reads, less than 0.5M umi-corrected reads, more than 1000 genes, and less than 20% mitochondrial read fraction were kept for downstream analysis. Doublet identification was performed using the python package scrublet, setting the doublet score at 0.15. First, single cell RNA seq analysis containing potential doublets as determined by scrublet was performed with scanpy in combination with custom-made code, and highly variable genes were identified as those with mean expression between 0.025 and 3; and minimum dispersion equal to 0.5. PCA analysis was performed using highly variable genes and the number of relevant PC was set as described in (Wagner, 2018). Cells were clustered using the Leiden algorithm with Manhattan distances on the relevant PC and 15 k-nearest neighbors and also using the Euclidean distances from the log-transformed gene expression table and the hierarchical clustering algorithm. All cells belonging to Leiden or Hierarchical clusters containing more than 50% predicted doublets were also labeled as potential doublets. Next, all cell barcodes labeled as doublets were removed from the dataset and the same single-cell RNA sequencing analysis pipeline described above was repeated.

#### Cell-cell communication analysis

The Cell-Cell communication assay was performed using the pre-processed single-cell data using Liana+ v.1.0.3 (BioRxiv: https://doi.org/10.1101/2023.08.19.553863) for each different sample. To obtain the cell-cell communication patterns we used the results from Liana+ and created an Intercellular Context Factorization using the Tensor-Cell2Cell algorithm v.0.7.3 (LIANA & Tensor-cell2cell tutorials: Combining LIANA and Tensor-cell2cell to decipher cell-cell communication across multiple samples. The Ligand-Receptor inference by Liana+ was run using the default parameters and no additional code was created. For the Tensor-Cell2Cell deconvolution, an elbow estimation was performed to then ran the pipeline using the optimal rank (rank = 9). No other changes were done.

### Quantification and statistical analysis

All results are represented as mentioned in figure legends, where the mean ± standard error of the mean (SEM) is shown. Each statistical test used for significant differences is written in the figure legend for each type of experiment. Data statistics were calculated in Prism8 (Graphpad). For statistical significance. ^∗^*p* < 0.05, ^∗∗^*p* < 0.01, ^∗∗∗^*p* < 0.001, n.s. not significant. For two conditions, t-test was used as a parametric or non-parametric test. For multiple independent groups, two-way ANOVA was performed as a parametric test. Tumor initiating capacity and statistical significance was calculated using ELDA software. For survival tests, Mantel-Cox statistics were used. For ROC Curve and contingency tables, Chi-square test was used.

## Supplemental information

Document S1. Figures S1–S8

Table S1. Upregulated and downregulated genes in IC mice obtained in the RNA-seq, related to Figure 1F Volcano plot of gene expression in all organs comparing IC and ID mice samples.

Table S2. LIANA network of micrometastatic samples (Ctrl/Tim3-KD) on the left, and macrometastatic samples (Ctrl/Tim3-KD) on the right for the interactors obtained in the factor 4, related to Figure 5 and supplementary Figure 5H LIANA network of micrometastatic samples (Ctrl/Tim3-KD) on the left, and macrometastatic samples (Ctrl/Tim3-KD) on the right for the interactors obtained in the factor 4.

Document S2. Article plus supplemental information
