# Supplementary material for: TIM3+ breast cancer cells license immune evasion during micrometastasis outbreak
Source: Cancer Cell. 2025 Aug 11;43(8):1549–1567.e9. doi: 10.1016/j.ccell.2025.06.015 (PMC12416865; doi:10.1016/j.ccell.2025.06.015)
Supplement: Document S1. Figures S1–S8 [file mmc1.pdf]

Cancer Cell, Volume *43*

Supplemental information

TIM3$^+$ breast cancer cells license immune evasion during micrometastasis outbreak

Catalina Rozalén, Irene Sangrador, Silvia Avalle, Sandra Blasco-Benito, Panagiota Tzortzi, María Sanz-Flores, José Ángel Palomeque, Pau Torren-Duran, Mariona Dalmau, Helena Brunel, Albert Coll-Manzano, Iván Pérez-Núñez, Tamara Martos, Sonia Servitja, Sandra Pérez-Buira, José Ignacio Chacón, Ángel Guerrero-Zotano, Eduardo Martínez de Dueñas, Yolanda Guillén, Laura Comerma, Begoña Bermejo, Anna Bigas, María Casanova-Acebes, Anna Alemany, Federico Rojo, Joan Albanell, and Toni Celià-Terrassa

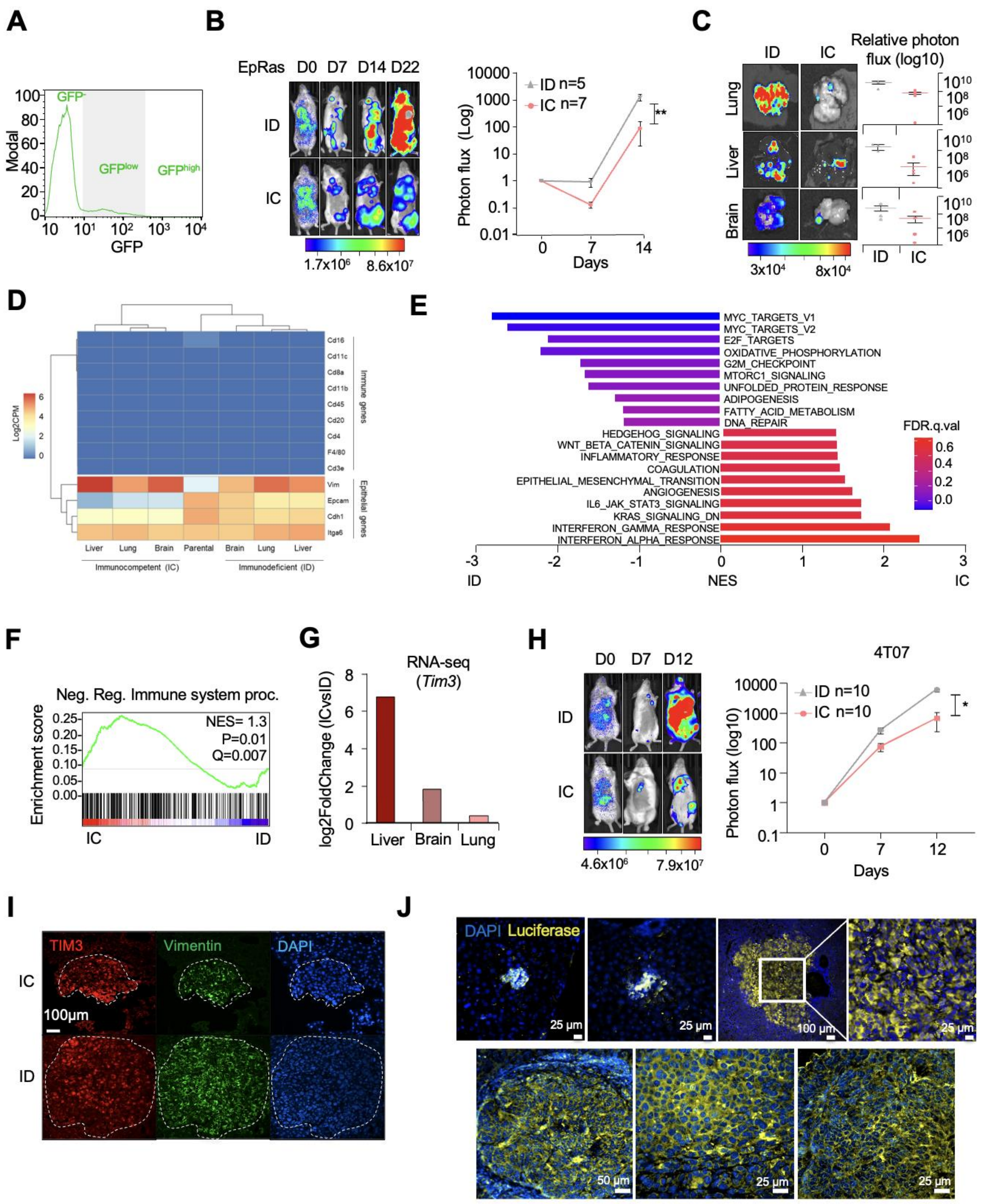

Supplementary Figure 1.

Supplementary Figure 1. Metastasis immunoediting uncovers TIM3⁺ metastatic cells, related to Figure 1.

(A) Flow cytometry histogram showing EpRas GFP intensity. GFP low cells were isolated to be used for the experimental metastasis procedures.

(B) Bioluminescence imaging (BLI) quantification and representative images of EpRas cell metastasis in immunocompetent (IC) Balb/c mice and immunodeficient (ID) NOD Scid Gamma (NSG) mice. Curves represent the mean of BLI signal per condition (n indicated in plot).

(C) BLI quantification of ex-vivo metastatic organs of EpRas metastasis.

(D) Heatmap of EpRas metastatic samples from all organs in IC and ID mice showing immune and epithelial gene values.

(E) GSEA enrichment analysis of the top significantly upregulated genes in the EpRas metastatic samples from all organs in IC (right) and ID (left) mice (n=3 independent biological replicates).

(F) GSEA of the indicated gene list with the ranked gene expression list of IC vs ID in all organs (lung, liver, brain) samples.

(G) Log2 fold change (FC) of *Tim3* mRNA levels of the EpRas RNA-seq metastatic samples separated by organ of metastasis (n=1).

(H) Bioluminescence imaging (BLI) quantification of 4T07 cells' metastasis in IC and ID mice. Curves represent the mean of BLI signal per condition (n indicated in plot). Statistics by one-way ANOVA test.

(I) Representative image of TIM3, vimentin, and DAPI immunofluorescent staining in 4T07 ID and IC liver metastasis. Scale bar, 100 µm. Dashed line delineates metastasis in hepatic stroma.

(J) Representative images of anti-luciferase and DAPI immunofluorescence staining in 4T07 IC liver micro- and macro-metastases. Scale bar indicated in each image.

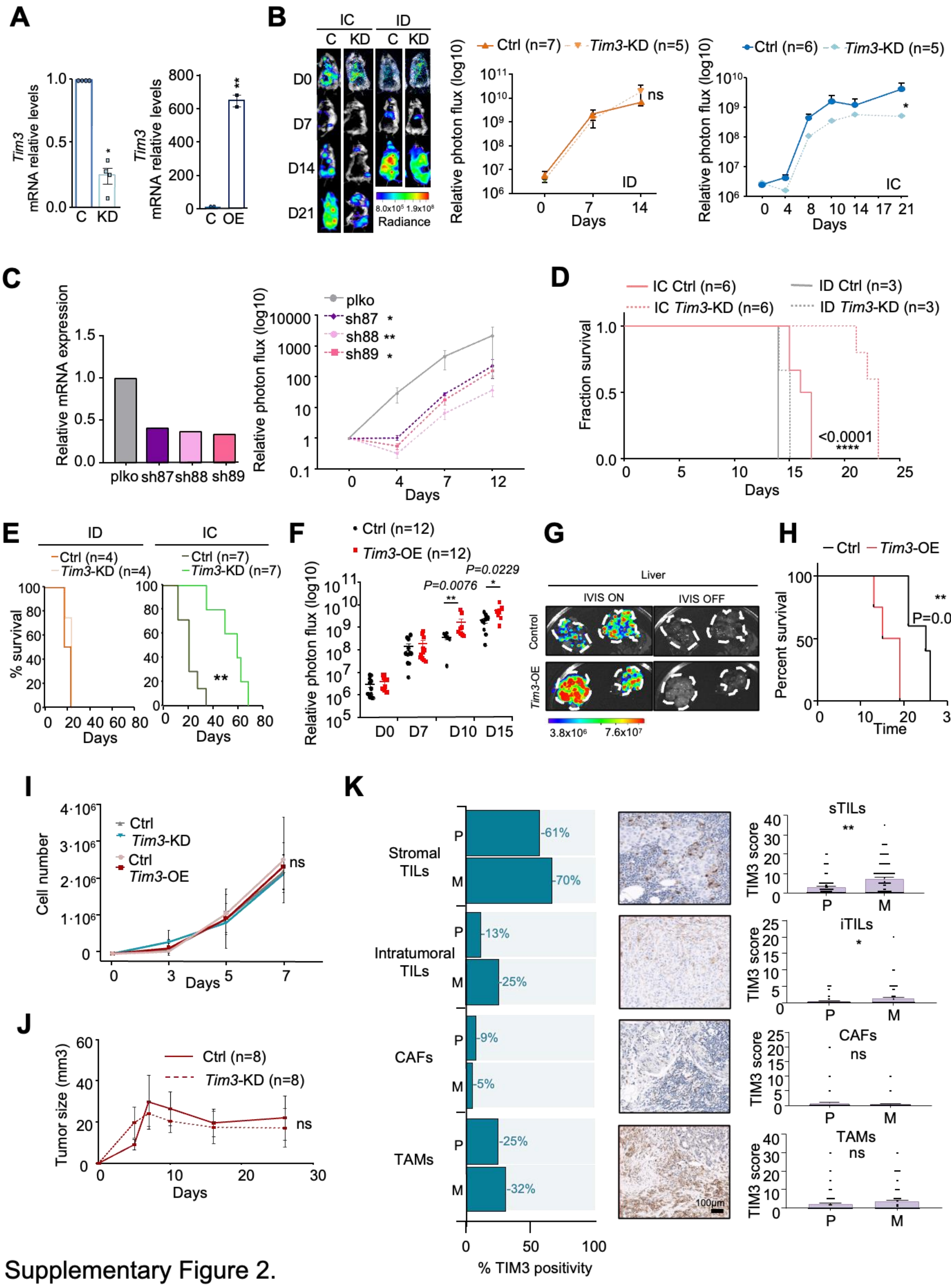

Supplementary Figure 2.

Supplementary Figure 2. TIM3 tumor cell-associated metastasis, related to Figure 2.

(A) RT-qPCR analysis of *Tim3* mRNA levels in 4T07 *Tim3*-KD (n=4 independent biological replicates). RT-qPCR analysis of *Tim3* overexpression (OE) cells (n=2 independent biological replicates).

(B) BLI representative images of metastatic progression in different conditions. BLI metastatic growth curve analysis after intracardiac injection of 4T07 control cells versus *Tim3*-KD cells in ID (NSG) and IC (Balb/c) mice. N of mice indicated in the figure. Statistics by one-way ANOVA test.

(C) RT-qPCR analysis of *Tim3* mRNA levels in 4T07 *Tim3*-KD using 3 additional different shRNAs (sh87, sh88, sh89). BLI metastatic growth curve analysis after intracardiac injection of 4T07 cells Control versus 3 different shRNAs for *Tim3*-KD.

(D) Kaplan-Meier survival plot after intracardiac injection of 4T07 control cells versus *Tim3*-KD unlabeled cells (neither GFP and luciferase) in ID (NSG) and IC (Balb/c) mice.

(E) Kaplan-Meier survival plot after intracardiac injection of AT3 control cells versus *Tim3*-KD cells in ID (NSG) and IC (C57Bl6) mice. N of mice indicated in the figure.

(F) BLI metastatic growth curve analysis after intracardiac injection of 4T07 control cells versus *Tim3*-OE cells in IC (Balb/c) mice. N of mice indicated in the figure.

(G) Representative BLI images of metastatic livers isolated from Ctrl and *Tim3*-OE mice. IVIS OFF (image before detection); IVIS ON (capturing luciferase signaling).

(H) Kaplan-Meier survival plot after intracardiac injection of 4T07 control cells versus *Tim3*-OE cells in IC (Balb/c) mice. N of mice indicated in panel F.

(I) In vitro cell proliferation assay representing cell number upon 7 days of Ctrl, *Tim3*-KD, Ctrl and *Tim3*-OE cells (n=3 independent biological replicates).

(J) Tumor volume of 4T07-Ctrl and 4T07-*Tim3*-KD primary tumors in Balb/c mice. N of mice indicated in the figure.

(K) Immunohistochemistry (IHC) quantification of TIM3 positivity for different cellular compartments. IHC TIM3 images and percentage scoring in stromal tumor-infiltrating lymphocytes (sTILs), intratumoral tumor-infiltrating lymphocytes (iTILs), cancer associated fibroblast (CAFs), and tumor associated macrophages (TAMs). Human tissue samples from primary (P) and metastatic (M) matched clinical samples from ConvertHER study (left panel). Each dot represents a patient (n=75 for each condition P and M). All data are represented as mean±SEM.

Statistical significance calculated by Log Rank (Mantel-Cox) test in panel D, E and H.

Statistical significance; *$p<0.05$, **$p<0.01$, ***$p<0.001$, by unpaired Student’s t-test in panel A and C, and paired Student’s t-test in panel F and I.

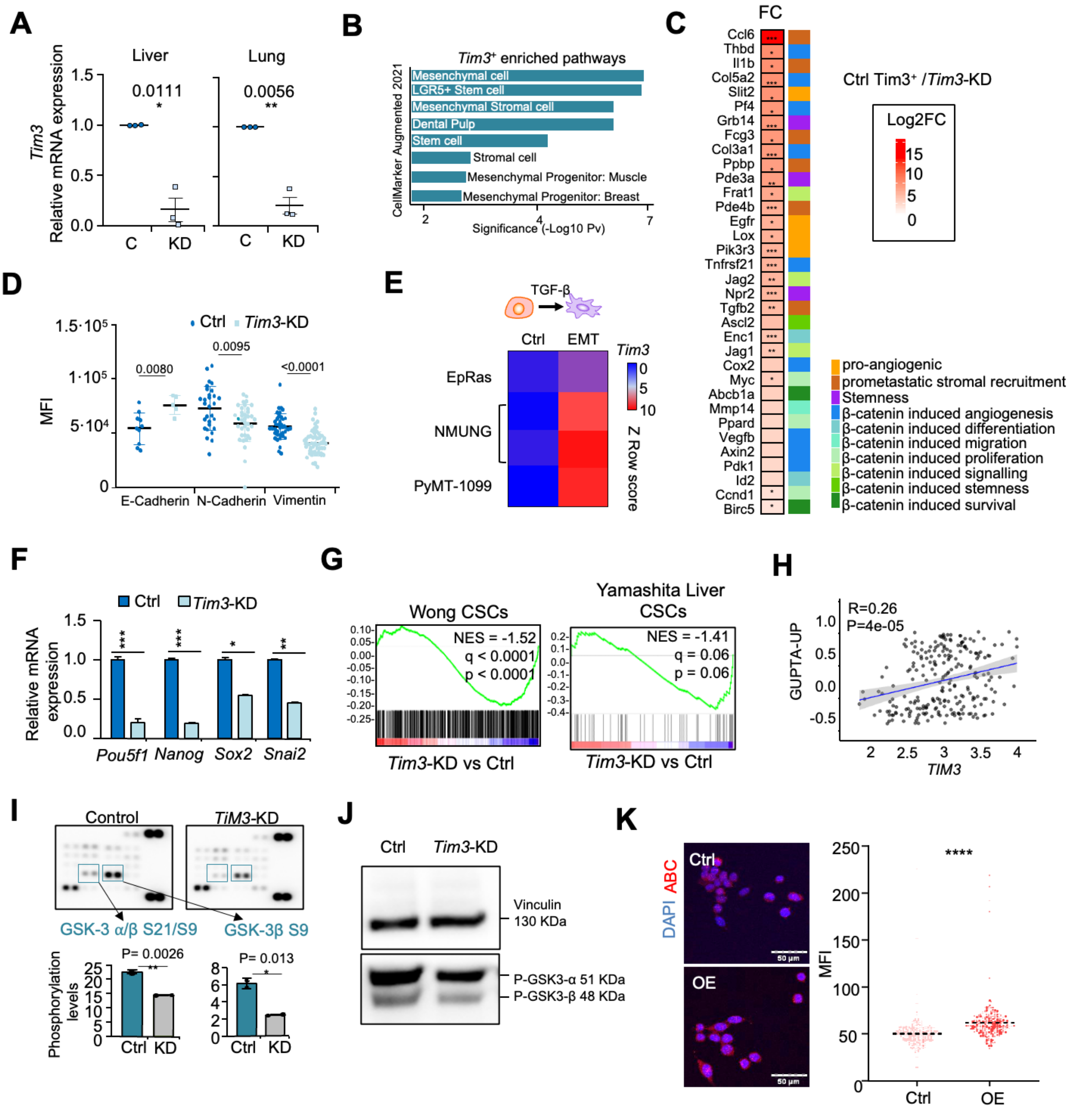

Supplementary Figure 3.

Supplementary Figure 3. TIM3 signaling in breast cancer cells and metastasis, related to Figure 3.

(A) RT-qPCR analysis of Ctrl and *Tim3*-KD 4T07 cell isolated from lung and liver metastasis (n=3 independent biological replicates); data represents mean±SEM.

(B) Cell type enrichment analysis of 4T07 TIM3 RNA-seq data (Ctrl vs *Tim3*-KD cells) using Enricher software interrogating the CellMarker Augmented 2021 dataset.

(C) Upregulated β-catenin target genes and functions in the Tim3 RNA-seq data heatmap (4T07 Ctrl and *Tim3*-KD metastasis).

(D) IF staining quantification of mean fluorescent intensity of EMT-like markers (E-cadherin, N-cadherin and vimentin) in ID and IC 4T07 liver metastasis (n=4 independent biological replicates). All data are represented as mean±SEM.

(E) Analysis of *Tim3* expression in EMT induction public datasets using different mammary cell lines. Corresponding study citations are indicated. From top to bottom, Celià-Terrassa et al., 2018; Grelet et al., 2021; Meyer-Schaller et al., 2019; Saxena et al., 2017.

(F) RT-qPCR analysis of Ctrl and *Tim3*-KD 4T07 cell (n=3 independent biological replicates); data represents mean±SEM.

(G) GSEA of transcriptomic ranked list of lung and liver metastatic organs in IC mice comparing Ctrl and *Tim3*-KD 4T07 cells interrogated with stem-like gene signatures.

(H) Rho correlation of *TIM3* and CSC *Up-regulated genes* in triple-negative breast cancer (TNBC) patients of the breast cancer TCGA dataset.

(I) Human phospho-kinase array for MDA-MB231 Ctrl and TIM3-KD cells. On the top, each dot of the membrane represents one specific phosphorylation of different kinases. On the bottom, the quantification of the phosphorylation levels in Ctrl and TIM3-KD conditions.

(J) Western blot of 4T07 mouse BC cells. Conditions: Ctrl and *Tim3*-KD. Blots show Vinculin, GSK3-α/β protein levels.

(K) Immunofluorescence of active β-catenin (ABC) and DAPI staining in 4T07-Ctrl and *Tim3*-OE cells. Quantification of the nuclear staining of ABC.

Statistical significance; *p<0.05, **p<0.01, ***p<0.001, by unpaired Student's t-test in panel A, C, D, F and K.

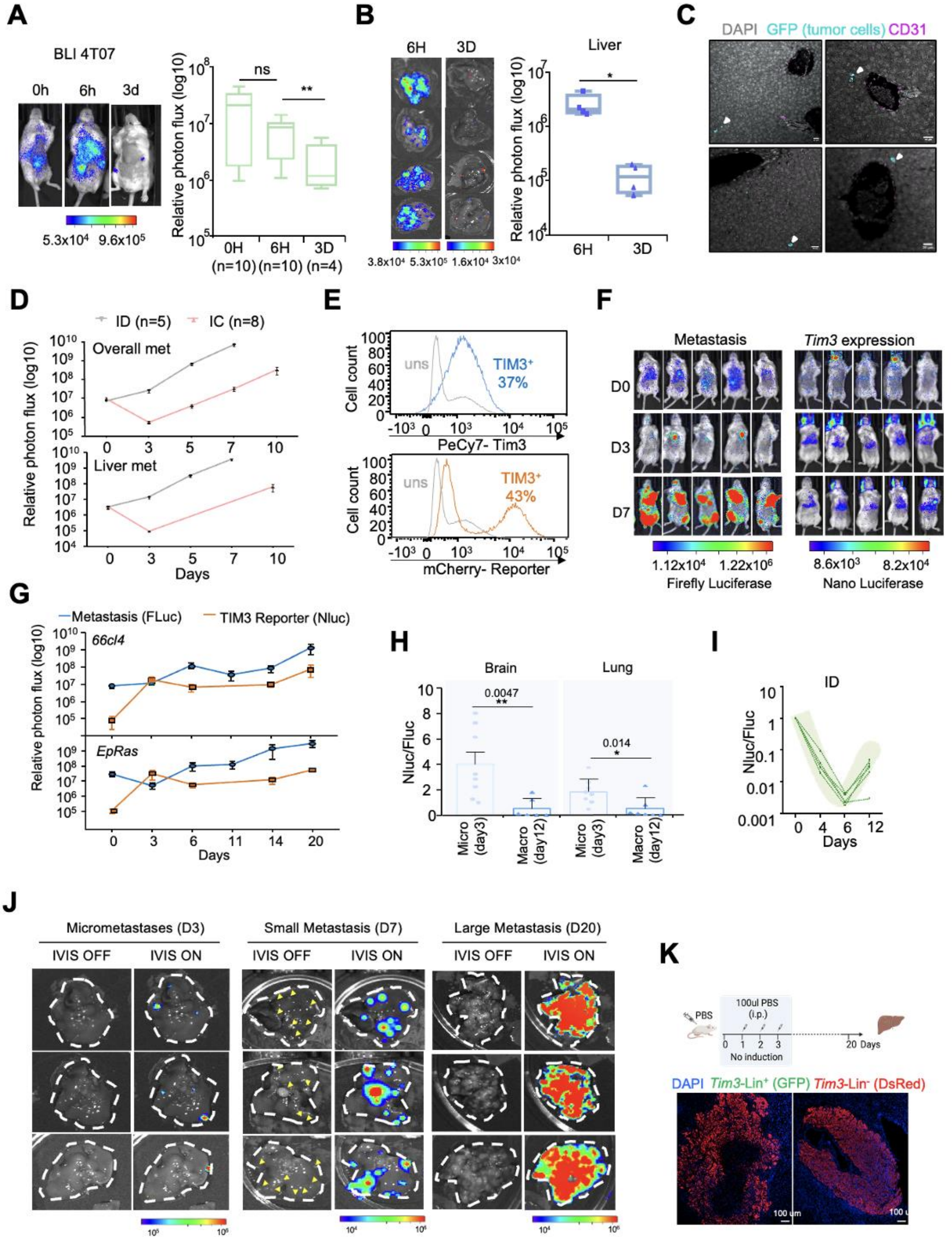

Supplementary Figure 4.

Supplementary Figure 4. Metastasis seeding and TIM3 dynamics, related to Figure 4.

(A) Bioluminescence (BLI) images of Balb/c mice injected with 4T07 cells at 0h, 6h, 3 days post injection. Box plots of BLI quantification at indicated timepoints.

(B) Bioluminescence (BLI) images of metastatic livers at 6h and 3d post injection. Box plots of BLI quantification at indicated timepoints.

(C) Immunofluorescence of tumor cells (anti-GFP) and blood vessels (anti-CD31) in liver sections after 6h of intracardiac injection. Scale bar, 20 µm.

(D) Bioluminescence (BLI) quantification of 4T07 cells in ID and IC hosts showing overall whole-body metastasis (top) and liver metastasis (bottom). Curves represent the mean of BLI signal per condition (n of mice indicated in the plot).

(E) Flow cytometry of mCherry intensity representing *Tim3* reporter status and TIM3 protein levels in 4T07 cells.

(F) Representative BLI images of Firefly luciferase (FLuc) and Nano luciferase (NLuc) measurements monitoring tumor bulk metastasis and *Tim3* expression, respectively.

(G) Experimental metastasis of 66cl4 (top) and EpRas (bottom) cells by i.c. injection and BLI analysis reporting tumor bulk whole body metastasis (FLuc) and *Tim3* expression (NLuc) in metastasis. Curves represent the mean of BLI signal per condition (n=8 mice).

(H) BLI ratio of NLuc/FLuc of brain and lung micrometastasis and macrometastasis timepoints after i.c. of 4T07 cells in IC (Balb/c) mice. Each point represents an individual mouse (8 independent mice per condition). $p<0.05$, $^{**}p<0.01$, $^{***}p<0.001$, by unpaired Student's t-test.

(I) BLI ratio dynamics of NLuc/FLuc of whole-body metastasis along days after i.c. of 4T07 cells in ID (NSG) mice. Each line represents an individual mouse.

(J) Ex vivo BLI detection of liver micrometastasis (3 days), small metastasis (7 days) and macrometastasis (12 days). IVIS OFF (image with camera off); IVIS ON (capturing luminiscence). Yellow arrows indicate metastatic nodules.

(K) *In vivo* test of no leakiness of the lineage system. Upon intracardiac injection, 100ul PBS were administered during the first 3 days. The system show no leakiness. Representative immunofluorescence images of livers sections showing $TIM3^-$ (red) metastasis in Balb/c mice after PBS treatment. Organs were harvested at day 20 of 4 independent experiments with a total of n = 25 metastases.

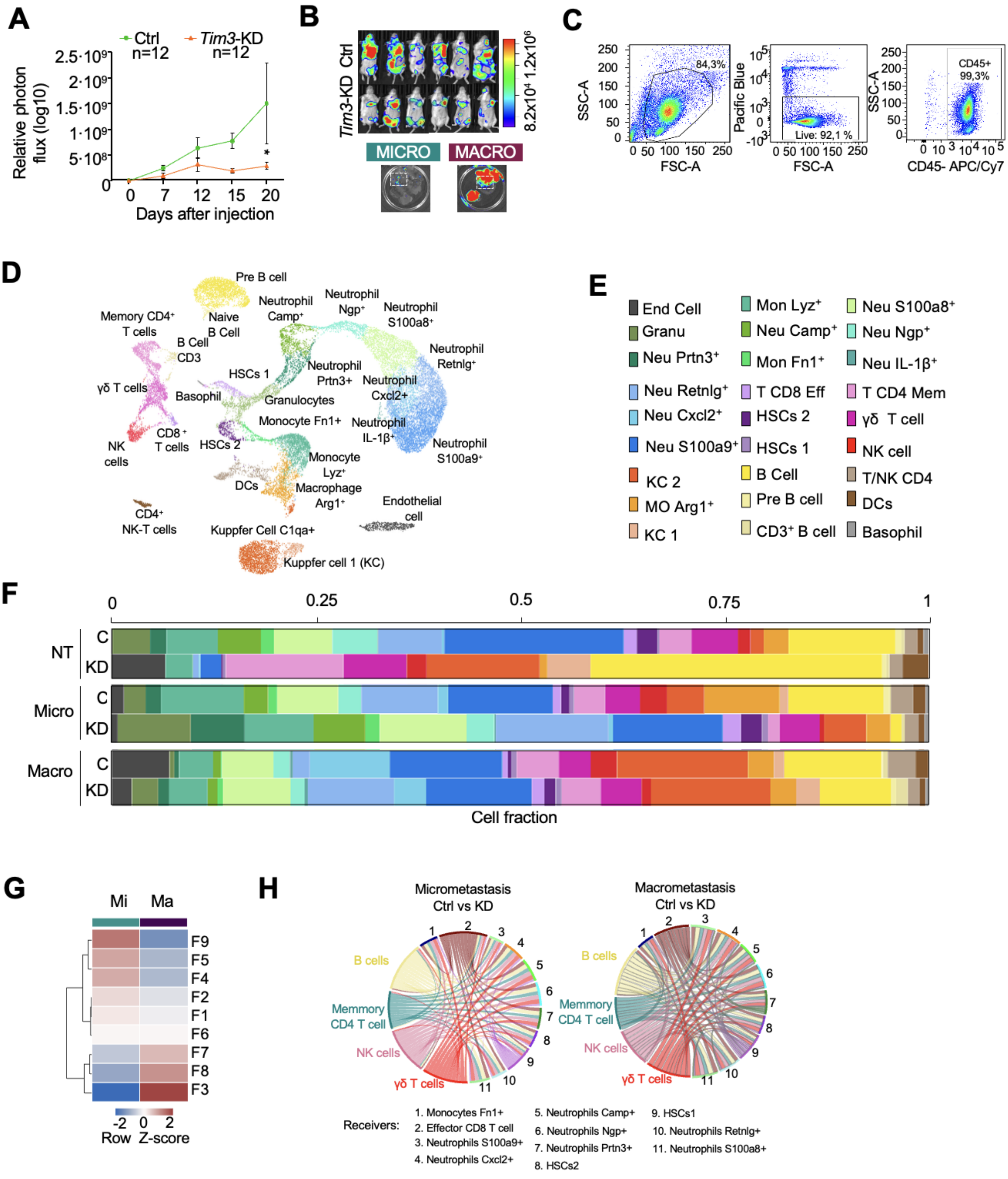

Supplementary Figure 5.

Supplementary figure 5. TIM3-tumor cell-mediated influence in metastasis immunity, related to Figure 5.

(A) Bioluminescence (BLI) quantification of metastasis of i.c. injected 4T07-Ctrl and *Tim3*-KD cells harvested and digested for the $CD45^+$ single cell-RNA-seq. Each bar represents the mean of 12 independent mice. Statistics by one-way ANOVA test.

(B) Representative BLI images of metastatic growth of 4T07 cells in IC mice. Bottom images show the criteria to dissect Micro- and Macro-metastatic samples.

(C) Flow cytometry gating strategy for $CD45^+$ immune cells sorting previously isolated from metastatic livers injected with Ctrl or *Tim3*-KD 4T07 cells in IC (Balb/c) mice.

(D) Single cell RNA-seq of $CD45^+$ cells isolated from liver metastasis. Uniform Manifold Approximation and Projection (UMAP) of $CD45^+$ immune cells isolated from liver metastasis after Ctrl and *Tim3*-KD i.c. injection of 4T07 tumor cells. Cells are colored according to Leiden clusters and are annotated for each immune cell subset.

(E) Leiden clusters annotated for each immune cell subset.

(F) Cell fraction color codes. Representation of the different immune subsets for the indicated conditions.

(G) Tensor cell-cell algorithm heatmap of Control samples from micro- and macro-metastatic samples representing the enrichment of interactors stratified into the different factors established. Intensity represents Z-row score.

(H) LIANA network of micrometastatic samples (Ctrl/*Tim3*-KD) on the left, and macrometastatic samples (Ctrl/*Tim3*-KD) on the right for the interactors obtained in the factor 4. Sender cells (colored clusters) and receiver cells (see legend). The width of connecting lines represents the strength of the interaction. Also see Table S2.

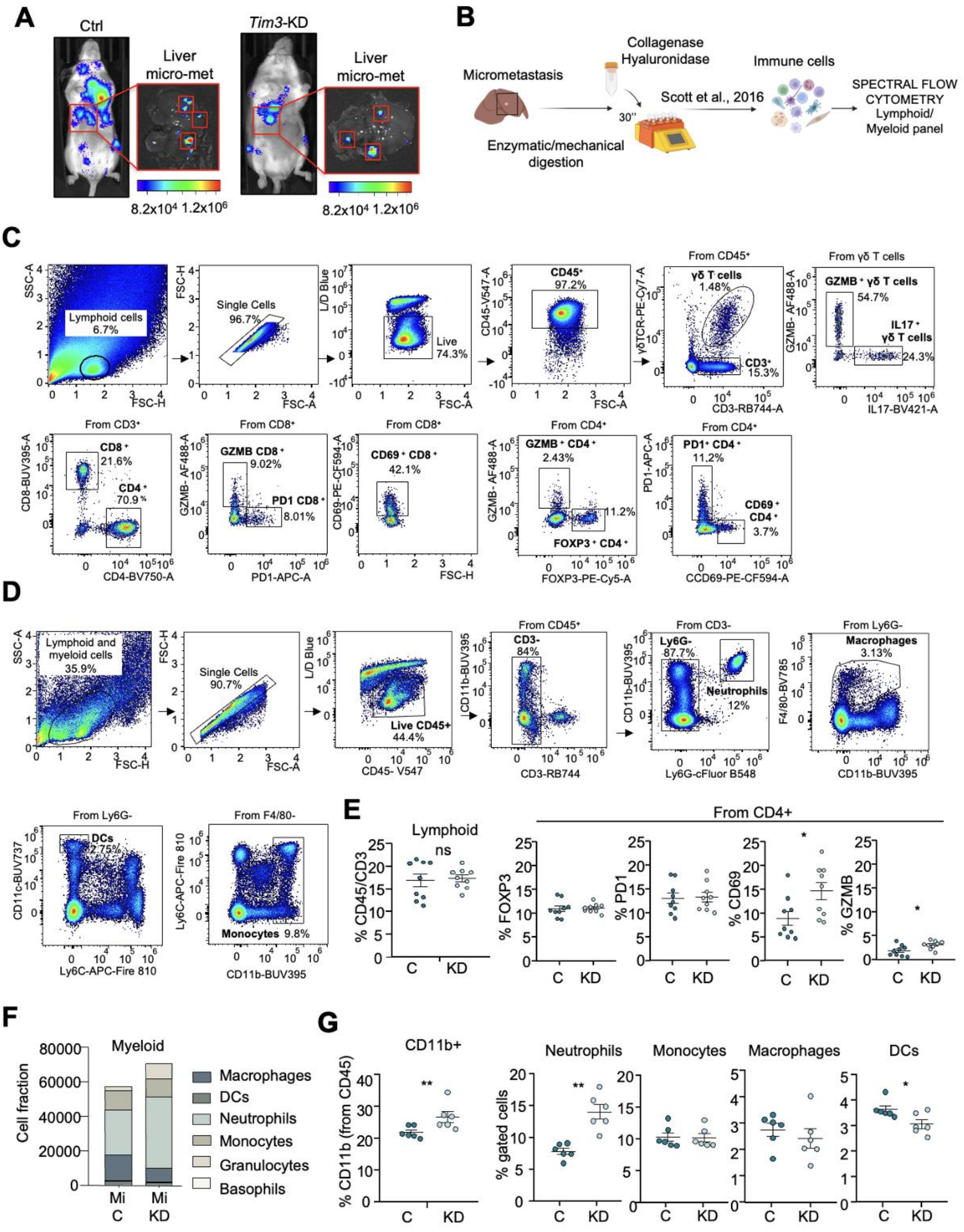

Supplementary Figure 6.

Supplementary figure 6. Flow cytometry validation of liver micrometastasis, related to Figure 5.

(A) Representative BLI imaging of 4T07 injected Balb/c mice at day 3-4 of micrometastasis. Red box represents micrometastasis detection and ex-vivo BLI of the liver to isolate a 5x5mm piece of tissue for immune cell isolation.

(B) Schematic representation of spectral flow cytometry validation. Upon BLI identification of micrometastasis at day 3-4, 5x5mm of liver are mechanically and enzymatically digested (see STAR Methods). Immune cells are finally purified and stained for spectral flow cytometry using myeloid and lymphoid panels.

(C) Gating strategy to determine lymphoid populations and their phenotypic markers of activation.

(D) Gating strategy to determine myeloid populations.

(E) Lymphoid quantification by flow cytometry of $CD3^+$ and $CD4^+$ T cells, quantification of FOXP3, PD1, CD69 and GZMB markers from Control and *Tim3*-KD liver micrometastasis. Each point represents an individual mouse (9 independent mice per condition).

(F) Cell fraction of myeloid populations from micrometastasis samples of the single-cell RNA-seq.

(G) Myeloid quantification by flow cytometry validation. Quantification of neutrophils, monocytes, macrophages and dendritic cells from Control and *Tim3*-KD liver micrometastasis. Each point represents an individual mouse (6 independent mice per condition).

p<0.05, **p<0.01, ***p<0.001, by unpaired Student's t-test in panel E and G.

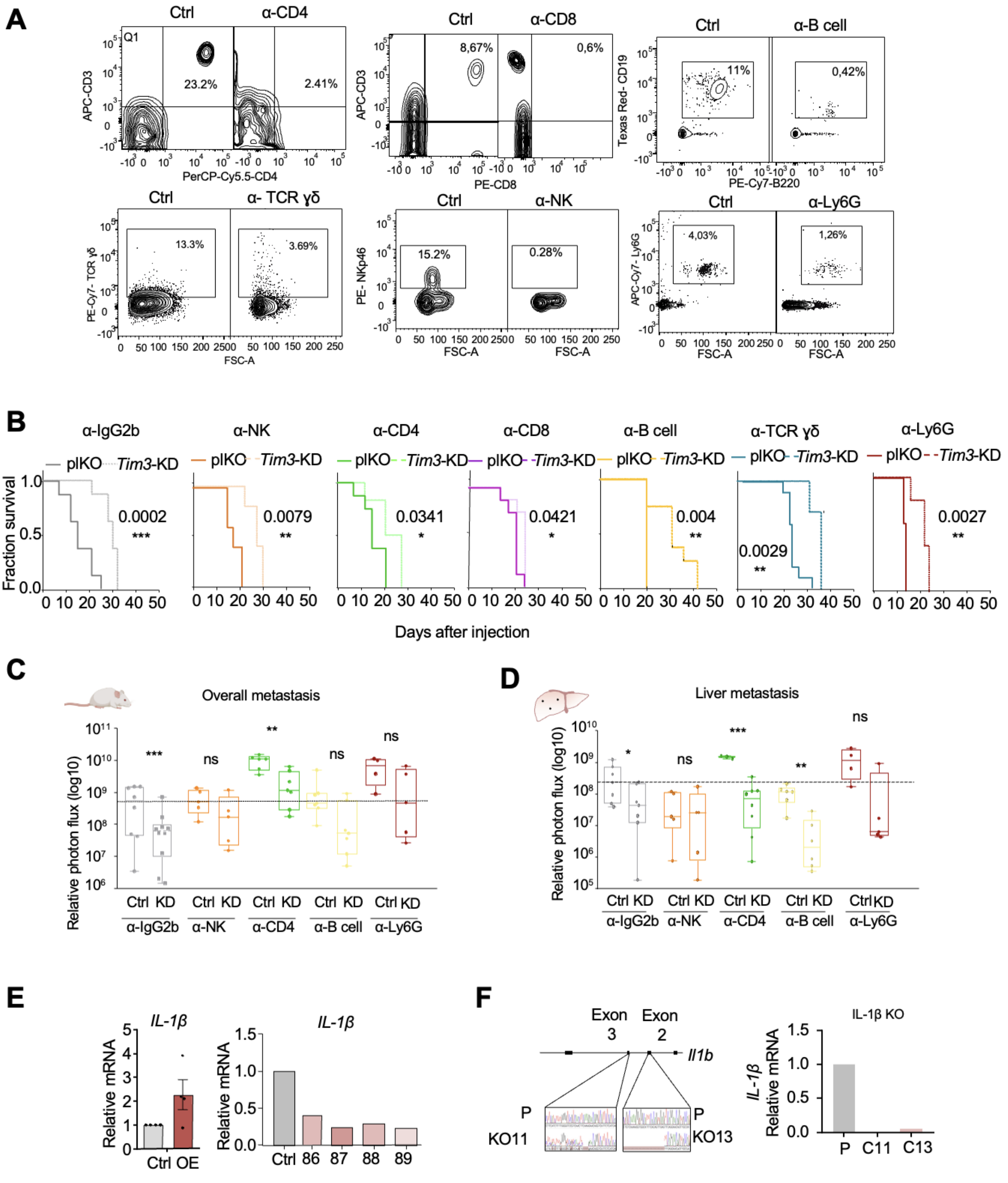

Supplementary Figure 7.

Supplementary figure 7. Functional analysis of immune cell populations, related to Figure 6.

(A) Flow cytometry validation of indicated antibody immune depletion or neutralization. Test performed in blood samples from Balb/c mice injected with 4T07 cells.

(B) Mice survival KM curves in 4T07 Ctrl and *Tim3*-KD cells metastasis during 40 days after neutralization of the indicated immune cell population. Each condition n=8 independent mice. The significance is calculated from Ctrl vs *Tim3*-KD; *p<0.05, **p<0.01, ***p<0.001, by Log Rank (Mantel-Cox) test.

(C) Box plots representing overall metastasis after indicated immune cell neutralization at day 13 after i.c. systemic delivery in Ctrl and *Tim3*-KD 4T07 cells. Data represents mean±SEM, each point represents an independent mouse.

(D) Box plots representing liver metastasis after indicated immune cell neutralization at day 13 after i.c. systemic delivery in Ctrl and *Tim3*-KD 4T07 cells. Data represents mean±SEM, each point represents an independent mouse.

(E) RT-qPCR analysis of *Il-1*β mRNA levels in 4T07 Ctrl and *Tim3*-OE on the left (n=4 independent biological replicates). On the right, *Il-1*β mRNA levels in 4T07 Ctrl and shRNAs for all *Tim3*-KD shRNAs used (sh86, sh87, sh88, sh89).

(F) CRISPR knock-out design of Il-1β gene in 4T07 cells. Clone 11 alters exon 3 of the Il1β gene. Clone 13 has a deletion in the Exon 2 of the Il1β gene. On the right, RT-qPCR analysis of Il1β mRNA levels of clone 11 and clone 13 compared to parental 4T07 cells.

p<0.05, **p<0.01, ***p<0.001, by unpaired Student's t-test in panel C and D.

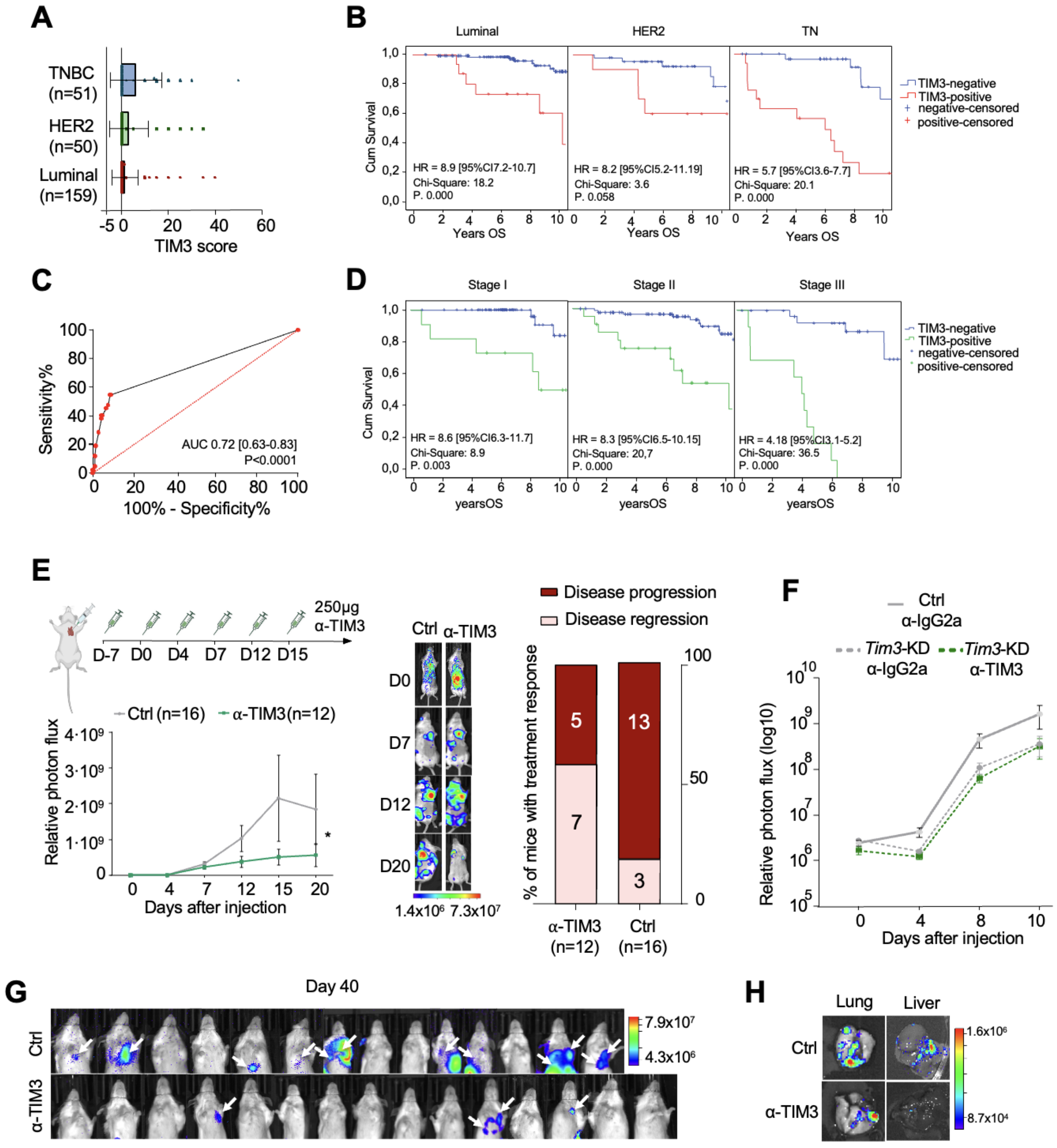

Supplementary Figure 8.

Supplementary figure 8. Tumor cell TIM3 clinical and preclinical data, related to Figure 7.

(A) TIM3 IHC percentage scoring in the different breast cancer subtypes: TNBC, HER2 and Luminal. Tissue microarrays (TMAs) with 260 breast cancer primary tumors from all subtypes.

(B) Overall survival (OS) Kaplan-Meier curves of primary tumor IHC epithelial-tumor $TIM3^+$ and $TIM3^-$ in the different breast cancer subtypes (Luminal, HER2, and TNBC). Statistical significance calculated by Log Rank (Mantel-Cox) for Chi-square and p-value.

(C) Univariate ROC curve analysis of relapsing and non-relapsing patients based on TIM3 positivity. Specificity and sensitivity are calculated based on the Area Under the Curve (AUC).

(D) Overall survival (OS) Kaplan-Meier curves of primary tumor IHC tumor $TIM3^+$ and $TIM3^-$ in stratified by disease stage I-III. Statistical significance calculated by Log Rank (Mantel-Cox) for Chi-square and p-value.

(E) Schematic representation of anti-TIM3 blockade treatment after intracardiac injection. Representative image of BLI progression. BLI quantification after 4T07 i.c. injection of whole-body metastasis with 250μg of anti-TIM3 therapy at the indicated periodicity. Bar plot representing response to the treatment at day 15. Disease progression (continuous increase of luciferase, higher than 10^7) and disease regression (reduced luciferase below 10^7).

(F) Bioluminescence (BLI) quantification of whole-body metastasis of i.c. injected 4T07-Ctrl, *Tim3*-KD cells treated with isotype (IgG2a), compared to anti-TIM3 antibody every 4 days using 250μg dosage. Each bar represents the mean of 11 independent mice.

(G) Upper body BLI images of spontaneous metastasis of Ctrl (IgG2a) and anti-TIM3 mice. Arrows indicate lung and liver metastasis. All mice represent 3 independent experiments using 4-5 mice per condition each. Total of 14 Ctrl mice, and 15 TIM3 blockade treated mice.

(H) Representative BLI image of *ex-vivo* metastatic organs to confirm the presence of lung and/or liver metastasis.
